# Cost-Effectiveness of Peer-Delivered Interventions for Cocaine and Alcohol Abuse among Women: A Randomized Controlled Trial

**DOI:** 10.1371/journal.pone.0033594

**Published:** 2012-03-20

**Authors:** Jennifer Prah Ruger, Arbi Ben Abdallah, Craig Luekens, Linda Cottler

**Affiliations:** 1 Department of Public Health, Yale University School of Medicine, New Haven, Connecticut, United States of America; 2 Washington University School of Medicine, St. Louis, Missouri, United States of America; Research and Development Corporation, United States of America

## Abstract

**Aims:**

To determine whether the additional interventions to standard care are cost-effective in addressing cocaine and alcohol abuse at 4 months (4 M) and 12 months (12 M) from baseline.

**Method:**

We conducted a cost-effectiveness analysis of a randomized controlled trial with three arms: (1) NIDA's Standard intervention (SI); (2) SI plus a Well Woman Exam (WWE); and, (3) SI, WWE, plus four Educational Sessions (4ES).

**Results:**

To obtain an additional cocaine abstainer, WWE compared to SI cost $7,223 at 4 M and $3,611 at 12 M. Per additional alcohol abstainer, WWE compared to SI cost $3,611 and $7,223 at 4 M and 12 M, respectively. At 12 M, 4ES was dominated (more costly and less effective) by WWE for abstinence outcomes.

**Conclusions:**

To our knowledge, this is the first cost-effectiveness analysis simultaneously examining cocaine and alcohol abuse in women. Depending on primary outcomes sought and priorities of policy makers, peer-delivered interventions can be a cost-effective way to address the needs of this growing, underserved population.

**Trial Registration:**

ClinicalTrials.gov NCT01235091

## Introduction

Over 9% of the total US population (23.1 million aged 12 or older) was in need of treatment for an illicit drug or alcohol use problem in 2008 [1]. Among females, the percentage of illicit drug users rose from 5.8% in 2007 to 6.8% in 2010; the number of users increased from 10.4% to 11.2% over the same period among males [1], [2]. Alcohol and illicit drug use were the third and ninth leading causes of preventable death in 2000, associated with 85,000 and 17,000 deaths, respectively [3]. Moreover, the economic costs of drug abuse in the US have increased 5.3% annually from 1992 to 2002, reaching a total of $180.9 billion, $16 billion of which were health-related [4]. Despite this rise in the costs of substance abuse, health care spending on substance abuse only rose 4.1% annually, whereas total health care spending rose by 6.5%.

A number of behavioral interventions have shown effectiveness in treating cocaine [5], [6] and alcohol abuse [7]. The Women Teaching Women intervention (WTW) was designed to reduce substance use and high-risk sexual behaviors through a holistic, peer-delivered intervention [8]. A randomized, controlled trial (RCT) examined three arms of WTW: (1) a modified National Institute on Drug Abuse (NIDA) Cooperative Agreement Standard Intervention (SI); (2) SI and a field-based Well Woman Exam (WWE); and (3) SI, WWE, and four Educational Sessions (4ES). The RCT took place in St. Louis, where, aside from alcohol, cocaine caused the greatest amount of treatment admissions in 2007 and remains a major drug problem [9], [10]. This study examined ten outcomes related to cocaine and alcohol use; a companion study analyzed the cost-effectiveness of these interventions for preventing, STDs and HIV [11]. With cost data from a previously published [12] micro-costing study of WTW conducted from the service provider and societal perspectives, we undertake an incremental cost-effectiveness analysis to determine the cost per outcome gained for each subsequent arm of the trial.

The literature surrounding cost-effectiveness analyses (CEAs) of prevention and treatment for cocaine and alcohol abuse continues to grow [13], [14], [15]. One article [16] examines the cost-effectiveness of a screening and brief intervention for alcohol abuse in a primary care setting. Others [15], [17], [18], [19] address the additional costs of voucher-based and prize-based contingency management programs above and beyond standard treatment for cocaine and opioid dependence. Two CEAs performed analyses alongside RCTs for alcohol treatment, one comparing social behavior and network therapy to motivational enhancement therapy [20], and another examining combined behavioral and pharmacotherapies [13]. An additional analysis illustrated the incremental cost-effectiveness of an enhanced, personalized HIV intervention compared to SI for preventing drug use [21].

Decision and policy makers need to be aware of interventions' impact on both cocaine and alcohol abuse because the two substances are often co-abused. Of the 1,648 cocaine-dependent patients in the national Drug Abuse Treatment Outcomes Studies (DATOS), 51% were also alcohol-dependent [22] and in a study of 158 alcohol-dependent outpatients, 62 of them also manifested cocaine abuse [23]. This study was conducted in accordance with the recommendations of the US Panel on Cost-Effectiveness in Health and Medicine [24].

## Methods

The protocol for this study and supporting CONSORT checklist and flow diagram are available as supporting information; see Checklist S1, Protocol S1, and Flow Diagram S1.

### Ethics Statement

This study has been approved by IRB Human Studies Committee at Washington University Hilltop (03-20), Washington University Medical Center (04-0285) and Yale University Human Investigation Committee (27312). The randomized controlled trial in the parent study has been registered with ClinicalTrials.gov and the registration number is 01235091. Data from the parent study was analyzed anonymously; written consent was obtained for the effectiveness data and for the cost data.

### Recruitment, Design, and Sample

Community health outreach workers recruited study participants from targeted recruitment zones, employing street-outreach methods based on prior work [25]. Eligibility criteria included: (1) being a woman over the age of 18; (2) reporting sexual activity in the prior four months; (3) using cocaine, heroin, amphetamines, or other injection drugs; and (4) residing in the St. Louis metropolitan area during the study period (2000–2006). Of the 501 women in the study, 420 participants received the standard intervention (SI) and completed baseline and both 4-months and 12-months endpoint assessments. They were randomized to one of three intervention conditions: SI alone (n = 135); WWE (n = 144); or 4ES (n = 141). Study participants were 90% racial/ethnic minorities. On average, they were 39 years old, had approximately 11 years of education, and worked for about 7 months when interviewed at baseline. The number of arrests ranged from an average of 4.9 for WWE participants to 9.4 for SI.

### Intervention Conditions and Costs

Interventions were administered at two community-based outreach centers shared with the St. Louis City Health Department. A peer facilitator – a female in drug recovery with at least one year's sobriety – conducted the SI, which included: (1) a 20-minute session for HIV pre-test counseling, blood collection, and administration of NIDA SI; [26] and (2) two weeks later, blood test results and HIV post-test counseling. The WWE intervention included an additional breast and pelvic examination with cervical cytological testing (Pap smear) provided by a nurse practitioner, who also obtained a short medical history. 4ES provided the additional opportunity of attending four educational sessions delivered by a peer facilitator paired with a health professional. The interactive sessions (1) were based on the Health Belief Model; (2) were focused on reducing unhealthy behaviors; and (3) employed a holistic approach, emphasizing health and nutrition, stress and coping, substance abuse, and HIV/AIDS.

The intervention costs employed in this analysis are based on a microcosting methodology, results of which are reported elsewhere [12].

### Effectiveness Measures

For cocaine use, the outcome measures include: occasions used (number of days used * episodes per day, past 30 days); episodes per day (past 30 days); cocaine free days (past 30 days); participants with negative urine tests; proportion of participants abstaining (past 30 days); and proportion of participants abstaining (past 4 months). For alcohol consumption, we examined the following outcomes: total drinks (past 7 days); drinks per day of the days participants drank alcohol (past 7 days); proportion of participants avoiding heavy drinking (past 7 days; “heavy drinking” defined as ≥4 drinks per day on the days participants drank alcohol); [6] and proportion of participants abstaining (past 30 days).

We estimated incremental effects by a two-step difference-in-differences approach in order to best compare results across trial arms. First, we calculated the difference in effectiveness from 4 months to baseline (4 M) and 12 months to baseline (12 M) within the same intervention. Second, differences within groups were compared to subsequent interventions to obtain the difference in differences, or incremental effects. In cases where a smaller mean from baseline indicates an intervention's effectiveness, the incremental effects were calculated to reflect the greater relative improvement by converting the change to a positive value.

### Incremental Cost-Effectiveness Analyses

We estimated the incremental cost-effectiveness of WWE compared to SI and 4ES compared to WWE by calculating the ratio of the difference in intervention costs (incremental costs) to the resulting incremental benefit (incremental effects). This calculation yields an incremental cost-effectiveness ratio (ICER), which provides the cost per obtaining an additional unit of outcome. An intervention is dominated if it is more costly and less effective than the alternative. Moreover, an intervention is extended dominated if the alternative is more costly, more effective and provides a lower ICER [13], [27]. An incremental cost-effectiveness analysis is appropriate in this study because each subsequent intervention adds significant components.

### Sensitivity Analyses

To examine the robustness of our findings and assess sampling variability, we conducted one- and two-way sensitivity analyses. One-way sensitivity analyses varied the costs and outcomes separately. Incremental costs of WWE were examined at $100, $200, $500 and $1,000 per participant. For 4ES, we evaluated alternative scenarios and their corresponding costs if: building rental/utilities decreased by 50% ($549) and 25% ($777); and, if each session had five people ($759) and three people ($1,233), rather than four [12]. We varied outcomes through statistically significant ranges of 5% intervals, determining when an intervention would become dominated or would no longer be dominated (“switching point”), depending on the base case. WWE and 4ES were also varied to achieve the following clinically significant levels: 12 and 15 occasions of cocaine use (“moderate to high” use); [28] 1 and 0.5 episodes of cocaine use per day (an approximate range of “moderate” use); 3 weeks of cocaine abstinence; [29] 85% cocaine free days (15% days used); [30] and, one and four drink(s) per day on the days participants drank alcohol (a range for “sporadic” and “heavy” drinkers) [31]. In two-way sensitivity analyses we varied these parameters simultaneously.

We constructed acceptability curves as an alternative to confidence intervals to address uncertainty in the results [32], [33], [34]. Selecting randomly with replacement (“bootstrapping”) from the base case samples yielded 1,000 new samples. From these new outcome means, 1,000 ICERs for each outcome measure were calculated and plotted as a function of willingness to pay (WTP) per additional abstainer. We analyzed the abstinence results because they are the most clinically meaningful outcome. The indicator of cocaine abstainers for the past 30 days provides a better comparison to alcohol abstainers, for which we only have data for the past 30 days. We chose the comparisons between WWE and SI once it became clear they provided better ICERs for both outcomes.

## Results

### Incremental Costs and Effects

**Table 1** presents treatment outcomes by treatment arm. **Table 2** reports the mean differences and percentage change in outcome measures by treatment arm. Each intervention achieved improvements above pre-treatment levels across all outcomes. **Tables 3** **and** **4** provide the incremental effects, indicating the improvements in Table 2 relative to the next least costly intervention. For cocaine abstainers, greater incremental gains were generally achieved at 4 M rather than 12 M. For example, both WWE and 4ES achieved incremental improvements at 4 M for cocaine abstainers in the past 30 days and past 4 months, whereas at 12 M only WWE for abstainers in the past 4 months achieved a positive incremental effect. This trend was not universal across all outcomes, however. WWE achieved better outcomes (Table 4) at 12 M than at 4 M for all alcohol outcomes except abstainers. The incremental costs per participant were $144 and $942 for WWE and 4ES, respectively [12].

**Table 1:** Effectiveness Measures by Treatment Group.

	SI	WWE	4ES
	(n = 135)	(n = 144)	(n = 141)
Treatment Outcome	Mean (s.d.)	Mean (s.d.)	Mean (s.d.)
**Cocaine Use**			
**Occasions** a **Used, past 30 days** b			
Pre-treatment	48.91 (96.2)	54.51 (104.9)	60.84 (144.1)
End of treatment, 4 mo	19.04 (41.2)	24.70 (69.6)	24.59 (63.3)
End of treatment, 12 mo	29.46 (62.6)	34.63 (71.8)	19.08 (38.7)
**Episodes per Day, past 30 days** b			
Pre-treatment	3.02 (3.8)	3.22 (3.8)	3.46 (5.7)
End of treatment, 4 mo	2.04 (3.3)	1.95 (3.2)	1.92 (3.6)
End of treatment, 12 mo	2.29 (4.9)	2.56 (5.2)	1.91 (2.7)
**Cocaine Free Days, past 30 days**			
Pre-treatment	19.21 (10.9)	19.33 (11.6)	19.66 (11.0)
End of treatment, 4 mo	25.03 (7.4)	24.97 (8.3)	24.63 (9.1)
End of treatment, 12 mo	22.31 (9.7)	22.06 (10.1)	24.26 (7.9)
**Participants with Negative Urine Tests** c			
Pre-treatment	21	19	14
End of treatment, 12 mo	34	32	26
**Abstaining,** d **past 30 days**			
Pre-treatment	0.13 (0.3)	0.13 (0.3)	0.14 (0.4)
End of treatment, 4 mo	0.43 (0.5)	0.44 (0.5)	0.50 (0.5)
End of treatment, 12 mo	0.33 (0.5)	0.33 (0.5)	0.32 (0.5)
**Abstaining,** d **past 4 months**			
Pre-treatment	0.07 (0.3)	0.06 (0.2)	0.08 (0.3)
End of treatment, 4 mo	0.33 (0.5)	0.34 (0.5)	0.41 (0.5)
End of treatment, 12 mo	0.28 (0.5)	0.31 (0.5)	0.30 (0.5)
**Alcohol Consumption**			
**Total Drinks, past 7 days** b			
Pre-treatment	19.26 (31.5)	22.02 (40.8)	22.41 (46.3)
End of treatment, 4 mo	11.75 (23.0)	15.66 (34.6)	12.00 (28.5)
End of treatment, 12 mo	16.40 (27.8)	14.01 (24.6)	13.03 (23.0)
**Drinks per Day,** e **past 7 days** b			
Pre-treatment	4.55 (5.9)	5.07 (7.5)	5.38 (7.9)
End of treatment, 4 mo	3.01 (4.8)	4.42 (8.2)	3.18 (5.2)
End of treatment, 12 mo	4.30 (7.3)	3.84 (5.4)	3.41 (4.9)
**Preventing Heavy Drinkers,** f **past 7 days** b			
Pre-treatment	0.59 (0.49)	0.59 (0.49)	0.57 (0.50)
End of treatment, 4 mo	0.69 (0.46)	0.66 (0.47)	0.67 (0.47)
End of treatment, 12 mo	0.60 (0.49)	0.63 (0.48)	0.62 (0.49)
**Abstaining,** g **past 30 days** b			
Pre-treatment	0.28 (0.45)	0.24 (0.43)	0.30 (0.46)
End of treatment, 4 mo	0.41 (0.49)	0.41 (0.49)	0.41 (0.49)
End of treatment, 12 mo	0.37 (0.49)	0.35 (0.48)	0.39 (0.49)

Abbreviations: SI, NIDA's Standard Intervention; WWE, SI plus Well Woman Exam; 4ES, SI, WWE plus four educational sessions.

a Occasions = days used * times per day.

b Missing data from 1–2 patients at some endpoints.

c Measured only at BL and 12 mo. The endpoint sample for each intervention included 121, 129 and 129 participants, respectively. Standard deviation not applicable here because value is number of participants.

d Proportion of participants abstaining from cocaine.

e Number of drinks per day, of the days participants drank alcohol.

f Proportion of participants avoiding heavy drinking. “Heavy drinking” defined as ≥4 drinks per day of the days participants drank alcohol.

g Proportion of participants abstaining from alcohol.

**Table 2:** Mean Differences and Percent Change by Treatment Group at 4 and 12 Months^a^.

	4 months			12 months		
Treatment Outcome	SI	WWE	4ES	SI	WWE	4ES
**Cocaine Use**						
Occasions Used^b^	−29.87 (−61.1%)	−29.81 (−54.7%)	−36.25 (−59.6%)	−19.45 (−39.8%)	−19.88 (−36.5%)	−41.76 (−68.6%)
Episodes per Day^c^	−0.98 (−32.5%)	−1.27 (−39.4%)	−1.54 (−44.5%)	−0.73 (−24.2%)	−0.66 (−20.5%)	−1.55 (−44.8%)
Days Free^d^	5.82 (30.3%)	5.64 (29.2%)	4.97 (25.3%)	3.10 (16.1%)	2.73 (14.1%)	4.60 (23.4%)
Negative Urine Tests^e^	N/A	N/A	N/A	13.00 (61.9%)	13.00 (68.4%)	12.00 (85.7%)
Abstaining, past 30 days^f^	0.30 (231%)	0.31 (238%)	0.36 (257%)	0.20 (154%)	0.20 (154%)	0.18 (129%)
Abstaining, past 4 mo^g^	0.26 (371%)	0.28 (467%)	0.33 (413%)	0.21 (300%)	0.25 (417%)	0.22 (275%)
**Alcohol Consumption**						
Total Drinks^h^	−7.51 (−39%)	−6.36 (−28.9%)	−10.41 (−46.5%)	−2.86 (−14.8%)	−8.01 (−36.4%)	−9.38 (−41.9%)
Drinks per Day^i^	−1.54 (−33.8%)	−0.65 (−12.8%)	−2.20 (−40.9%)	−0.25 (−5.5%)	−1.23 (−24.3%)	−1.97 (−36.6%)
Preventing Heavy Drinkers^j^	0.10 (16.9%)	0.07 (11.9%)	0.10 (17.5%)	0.01 (1.7%)	0.04 (6.8%)	0.05 (8.8%)
Abstaining^k^	0.13 (46.4%)	0.17 (70.8%)	0.11 (36.7%)	0.09 (32.1%)	0.11 (45.8%)	0.09 (30.0%)

Abbreviations: SI, NIDA's Standard Intervention; WWE, SI plus Well Woman Exam; 4ES, SI, WWE plus four educational sessions.

a Values represent differences and percentage change (parentheses) within groups from baseline. Due to rounding, calculations may not exactly reflect data from Table 1.

b Number of occasions used cocaine (number of days used * times per day); negative number indicates improvement.

c Number of episodes used cocaine per day; negative number indicates improvement.

d Number of days free from cocaine; positive number indicates improvement.

e Number of negative urine tests; positive number indicates improvement. We examined baseline and incremental data, but did not perform an incremental cost-effectiveness analysis on this outcome due to missing data.

f Proportion of participants abstaining from cocaine, past 30 days; positive number indicates improvement.

g Proportion of participants abstaining from cocaine, past 4 months; positive number indicates improvement.

h Total number of alcoholic drinks, past 7 days; negative number indicates improvement.

i Mean number of alcoholic drinks per day, of the days participants drank alcohol, past 7 days; negative number indicates improvement.

j Proportion of participants avoiding heavy drinking, past 7 days; “Heavy drinking” defined as ≥4 drinks per day of the days participants drank alcohol; positive number indicates improvement.

k Proportion of participants abstaining from alcohol, past 30 days; positive number indicates improvement.

**Table 3:** Incremental Cost-Effectiveness Analyses: Cocaine Outcomes^a^.

	Incremental Costs	Incremental Effects^b^					ICER (ΔC/ΔE, $)				
	Δ Costs per participant	Occasions Used^c^	Episodes per Day^d^	Days Free^e^	Abstaining, past 30 days^f^	Abstaining, past 4 months^g^	Cost per Occasion Averted^h^	Cost per Episode Reduced^i^	Cost per Cocaine Free Day^h^	Cost per Abstainer, past 30 days	Cost per Abstainer, past 4 months
**Treatment Groups**											
**BL to 4 mo**											
SI	--	--	--	--	--	--	--	--	--	--	--
SI+WWE	$144.45	−0.06	0.29	−0.18	0.01	0.02	D	$498.10	D	$14,445.00	$7,222.50
SI+WWE+4ES	$942.30	6.44	0.27	−0.67	0.05	0.05	$146.32	$3,490.00	D	$18,846.00	$18,846.00
**BL to 12 mo**											
SI	--	--	--	--	--	--	--	--	--	--	--
SI+WWE	$144.45	0.43	−0.07	−0.37	0	0.04	ED	D	D	D	$3,611.25
SI+WWE+4ES	$942.30	21.88	0.89	1.87	−0.02	−0.03	$43.07	$1,058.76	$503.90	D	D

Abbreviations: SI, NIDA's Standard Intervention; WWE, SI plus Well Woman Exam; 4ES, SI, WWE plus four educational sessions; ICER, incremental cost effectiveness ratio, which is the difference in cost divided by the difference in effectiveness as compared with the next least costly intervention, and indicates cost per additional outcome achieved; BL, baseline; D, dominated, which indicates that the intervention is more costly and less effective than the alternative; ED, extended dominated, which indicates that the next alternative is more costly, more effective and has a better ICER.

a Costs from Ruger et al., 2010. Due to rounding, calculations may not exactly reflect data from Table 2. ICERs did not need to be adjusted because the intervention has a sufficiently long follow up time period.

b Positive number indicates incremental improvement. Each value is the difference of the differences from Table 2.

c Number of occasions used cocaine, past 30 days (number of days used * times per day).

d Number of episodes used cocaine per day, past 30 days.

e Number of cocaine free days, past 30 days.

f Proportion of participants abstaining from cocaine, past 30 days.

g Proportion of participants abstaining from cocaine, past 4 months.

h Past 30 days.

i Per day, past 30 days.

**Table 4:** Incremental Cost-Effectiveness Analyses: Alcohol Outcomes^a^.

	Incremental Costs	Incremental Effects^b^				ICER (ΔC/ΔE, $)			
	Δ Costs per participant	Total Drinks^c^	Drinks per Day^d^	Preventing Heavy Drinkers^e^	Abstaining^f^	Cost per Drink Avoided^g^	Cost per Reduced Drink per Day^g^	Cost per Heavy Drinker Prevented^h^	Cost per Abstainer^i^
**Treatment Groups**									
**BL to 4 mo**									
SI	--	--	--	--	--	--	--	--	--
SI+WWE	$144.45	−1.15	−0.89	−0.03	0.04	D	D	D	$3,611.35
SI+WWE+4ES	$942.30	4.05	1.55	0.03	−0.06	$232.67	$607.94	$31,410.00	D
**BL to 12 mo**									
SI	--	--	--	--	--	--	--	--	--
SI+WWE	$144.45	5.15	0.98	0.03	0.02	$28.05	$147.40	$4,815.00	$7,222.50
SI+WWE+4ES	$942.30	1.37	0.74	0.01	−0.02	$687.81	$1,273.38	$94,230.00	D

Abbreviations: SI, NIDA's Standard Intervention; WWE, SI plus Well Woman Exam; 4ES, SI, WWE plus four educational sessions; ICER, incremental cost effectiveness ratio, which is the difference in cost divided by the difference in effectiveness as compared with the next least costly intervention, and indicates cost per additional outcome achieved; BL, baseline; D, dominated, which indicates that the intervention is more costly and less effective than the alternative.

a Costs from Ruger et al., 2010. Due to rounding, calculations may not exactly reflect data from Table 2. ICERs did not need to be adjusted because the intervention has a sufficiently long follow up time period.

b Positive number indicates incremental improvement. Each value is the difference of the differences from Table 2.

c Total number of alcoholic drinks, past 7 days.

d Mean number of alcoholic drinks per day, of the days participants drank alcohol, past 7 days.

e Proportion of participants avoiding heavy drinking, past 7 days. “Heavy drinking” defined as ≥4 drinks per day of the days participants drank alcohol.

f Proportion of participants abstaining from alcohol, past 30 days.

g Past 7 days.

h Past 7 days. “Heavy drinker” includes participants who drink ≥4 drinks per day of the days participants drank alcohol.

i Past 30 days.

### Incremental Cost-Effectiveness Analyses: Cocaine Outcomes

Columns 7–11 of **Table 3** report the incremental cost-effectiveness ratios across all cocaine outcomes at 4 M and 12 M. Comparing WWE to SI at 4 M, the cost per additional abstainer (past 30 days) was $14,445, but WWE was dominated by SI at 12 M. In contrast, at 4 M the ICER for WWE compared to SI of abstainers in the past 4 months was $7,223, but only $3,611 at 12 M. To obtain an additional abstainer (past 4 months) at 4 M, WWE compared to SI cost $11,624 less than 4ES compared to WWE. Furthermore, an additional abstainer for the past 30 days at 4 M cost $4,401 less for WWE compared to SI than for 4ES compared to WWE.

The interventions generated lower ICERs, when not dominated, for additional occasion averted, episode reduced per day, and cocaine free day than for the abstinence outcomes. Moreover, 4ES compared to WWE had lower ICERs for the non-abstinence outcomes at 12 M than at 4 M.

### Incremental Cost-Effectiveness Analyses: Alcohol Outcomes

Columns 6–9 of **Table 4** report the incremental cost-effectiveness ratios across all alcohol outcomes at 4 M and 12 M. For cost per abstainer, WWE was cost-effective compared to SI, with an ICER of $3,611 at 4 M and $7,223 at 12 M, whereas WWE dominated 4ES at both 4 M and 12 M. Conversely, WWE was dominated by SI at 4 M for all other alcohol outcomes (drinks, drinks per day, and heavy drinkers prevented).

At 4 M, 4ES compared to WWE cost $233 and $608 to avoid an additional drink and to reduce an additional drink per day, respectively. Moreover, an additional heavy drinker was prevented by 4ES compared to WWE at a cost of $31,410.

At 12 M, only 4ES compared to WWE for the cost per abstainer was dominated. The cost per heavy drinker prevented for 4ES compared to WWE provided the largest ICER ($94,230). The smallest ICER was the cost per additional drink avoided comparing WWE to SI ($28), whereas comparing 4ES to WWE on the same outcome yielded an ICER of $688. WWE compared to SI cost $4,815 to prevent an additional heavy drinker. For the cost per reduced drink per day, ICERs were $147 for WWE compared to SI and $1,273 for 4ES compared to WWE.

### Sensitivity Analyses

#### One-way sensitivity analyses

Varying the costs per participant changed the ICERs proportionally (Table S1). At 4 M, for cocaine abstainers (past 4 months), the ICER for WWE compared to SI ranged from $5,000 to $50,000 (base case ICER: $7,223) at $100 to $1,000 per participant, whereas comparing 4ES to WWE at costs that varied from $549 to $1,233 yielded ICERs from $10,980 to $24,660 (base case: $18,846). At 4 M, for alcohol abstainers, WWE ranged from $2,500 to $25,000 (base case: $3,611) at $100 to $1,000 per participant and at 12 M the equivalent cost changes yielded an ICER range of $5,000 to $50,000 (base case: $7,223). For WWE at incremental costs of $500 or $1,000 (base case: $144.45), excluding dominated options, only cocaine abstainers in the past 30 days at 4 M yielded ICERs above $50,000. Generating the smallest ICERs, the cost per cocaine occasion averted for 4ES at 12 M varied from $25 to $56.

Varying the effectiveness parameters across statistically (**Table 5**) and clinically significant ranges (**Table 6**) indicated the sensitivity of each finding. Some outcomes required greater changes than others to achieve a switching point. For example, 4ES required a 115% effectiveness decrease at 12 M and a 30% decrease at 4 M in reducing occasions of cocaine use and a 50% decrease at 12 M and a 15% decrease at 4 M in reducing an episode of cocaine use per day to become dominated by WWE. If WWE obtained 10% fewer cocaine abstainers (past 4 months), it was dominated by SI at 4 M but was still cost-effective at 12 M ($16,050; Table S2) and did not become dominated by SI until its effectiveness was decreased by 15%. Moreover, 4ES remained cost-effective in reducing a drink per day at 4 M up to the point at which its effectiveness decreased by 50%, while WWE at 4 M required a 25% increase in effectiveness to eliminate SI domination.

**Table 5:** One-Way Sensitivity Analyses of Switching Points: Outcomes.

	ICER (ΔC/ΔE, $), 4 mo		ICER (ΔC/ΔE, $), 12 mo	
Parameter Varied^a^	B-A	C-B	B-A	C-B
**Cocaine Use**				
Occasions^b^ Used, past 30 days	+5% ($123)	−30%	−5%	−115%
Episodes per Day, past 30 days	−15%	−15%	+5% ($2,491)	−50%
Cocaine Free Days, past 30 days	+5% ($135)	+5% ($1,678)	+5% ($197)	−10%
Abstaining, past 30 days	−5%	−10%	+5% ($8,755)	+10% ($78,525)
Abstaining, past 4 months	−10%	−15%	−15%	+15% ($62,820)
**Alcohol Consumption**				
Total Drinks, past 7 days	+10% ($347)	−35%	−40%	−15%
Drinks per Day, past 7 days	+25% ($672)	−50%	−30%	−25%
Preventing Heavy Drinkers,^c^ past 7 days	+5% ($48,150)	−5%	−5%	−5%
Abstaining, past 30 days	−10%	+15% ($628,200)	−10%	+10% ($49,595)

Abbreviations: ICER, incremental cost effectiveness ratio, which is the difference in cost divided by the difference in effectiveness as compared with the next least costly intervention and indicates cost per additional outcome achieved; A, SI intervention; B, WWE intervention; C, 4ES intervention.

Table S2 includes complete results of ICERs at 5% increments en route to the switching points.

a Percentage varied indicates percentage change of base case value for more costly alternative only, whereas base case of its comparator remain the same. A negative percentage change indicates less effective intervention than the base case, which may or may not indicate a mean lower than the base case (see Table 2). A positive percentage change indicates a more effective intervention than base case. Included in parentheses is the corresponding ICER.

b Occasions = days used * times per day.

c “Heavy drinker” includes participants who drink ≥4 drinks per day of the days participants drank alcohol.

**Table 6:** One-Way Sensitivity Analyses of Clinically Significant Outcomes.

	ICER (ΔC/ΔE, $), 4 mo		ICER (ΔC/ΔE, $), 12 mo	
Parameter Varied^a^	B-A	C-B	B-A	C-B
**Cocaine Use**				
Occasions^b^ Used, past 30 days - 12	$11	$50	$6	$33
Occasions^b^ Used, past 30 days - 15	$15	$59	$7	$36
Episodes per Day, past 30 days - 1	$116	$792	$97	$524
Episodes per Day, past 30 days - 0.5	$83	$558	$73	$410
Cocaine Free Days, past 30 days - 3 weeks	$51	$349	$26	$168
Cocaine Free Days, past 30 days - 85% free	$413	$4,712	$47	$303
**Alcohol Consumption**				
Drinks per Day, past 7 days - 4	D	$1,291	$176	$6,282
Drinks per Day, past 7 days - 1	$57	$253	$38	$299

Abbreviations: ICER, incremental cost effectiveness ratio, which is the difference in cost divided by the difference in effectiveness as compared with the next least costly intervention and indicates cost per additional outcome achieved; A, SI intervention; B, WWE intervention; C, 4ES intervention.

a Indicates the outcome achieved in the analysis.

b Occasions = days used * times per day.

Conversely, an increase or decrease of as low as 5% revealed a switching point for other cocaine and alcohol outcomes. For example, WWE was dominated by SI at 4 M and 12 M for cocaine free days in the base case; however, a 5% increase in effectiveness for WWE eliminated domination and yielded an ICER of $135 at 4 M and $197 at 12 M.

#### Two-way sensitivity analyses

We also determined the impact of more than one factor on results (Table S3).

For the cost per additional cocaine abstainer (past 30 days),4ES at 4 M yielded ICERs ranging from $5,490 to $12,330 at incremental costs of $549 to $1,233 per participant and a 10% increase in effectiveness. For cocaine free days, a 5% increase in effectiveness and cost range of $100 to $1,000 per participant for WWE compared to SI yielded an ICER range of $94 to $936 at 4 M and $136 to $1,364 at 12 M (base case: dominated). For alcohol outcomes, relative to SI, WWE would cost $51,282 per additional alcohol abstainer at 4 M if the effectiveness of WWE decreased by 5% and incremental costs increased to $1,000 per participant.

#### Threshold Analyses

**Figure 1:**
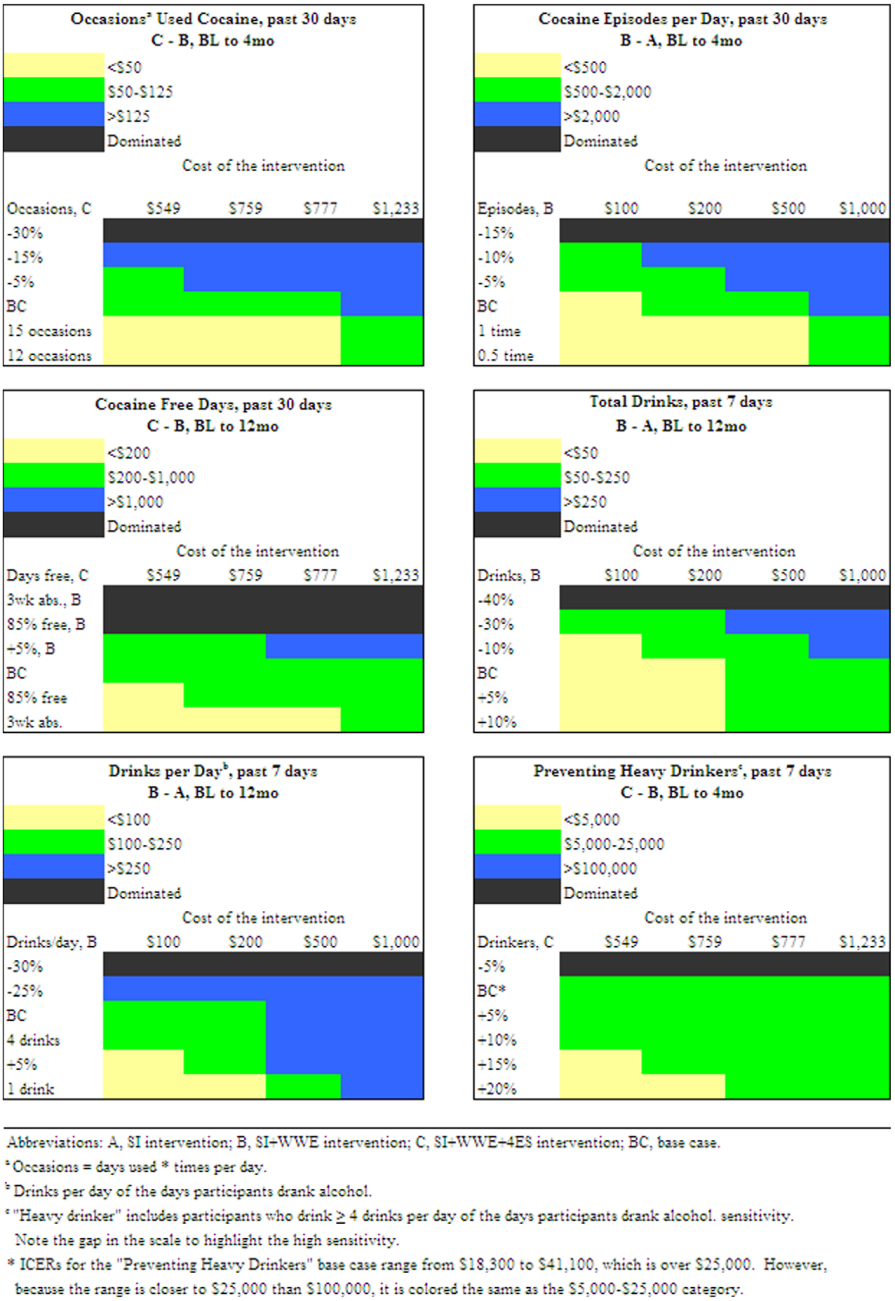
Two-way Sensitivity Analyses on Cost and Effectiveness.

**Figure 1** illustrates changes in ICERs relative to changes in costs and effectiveness in a stepwise fashion. For cocaine episodes per day, WWE was dominated by SI with a 15% decrease in effectiveness for WWE, but yielded an ICER under $2,000 with an incremental cost of $100 and decrease in effectiveness of 10%. At incremental costs of $500, WWE was cost-effective compared to SI at as large a decrease as 10% in effectiveness, yielded an ICER under $2,000 at base case effectiveness, and under $500 if WWE achieved a mean of 1 episode per day. 4ES proved highly sensitive for preventing heavy drinkers with an ICER under $25,000 across all costs with a 5% increase in effectiveness.

#### Acceptability Curves

**Figure 2:**
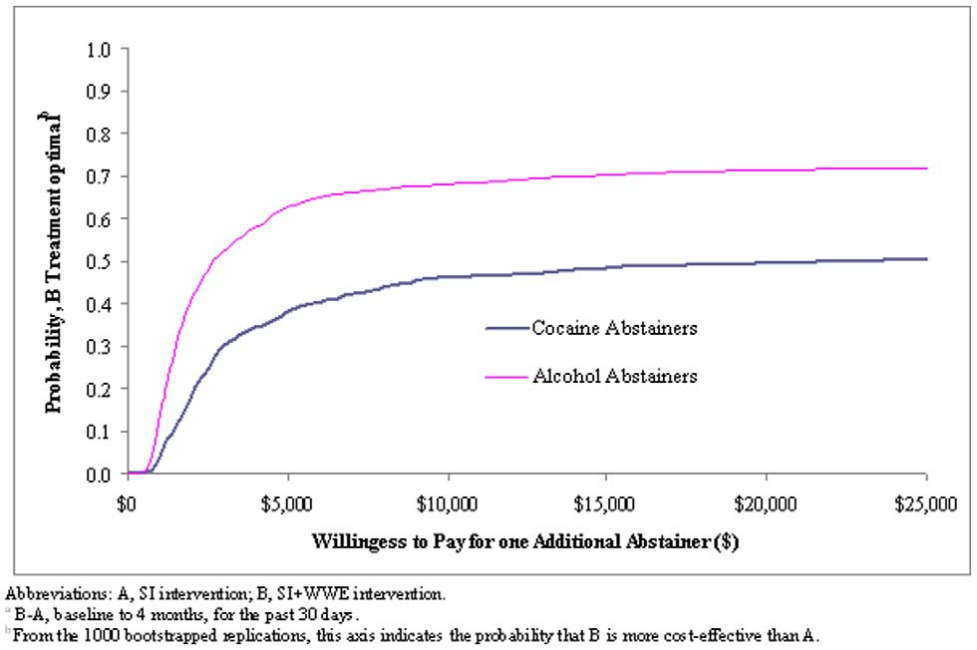
Acceptability Curves.

**Figure 3:**
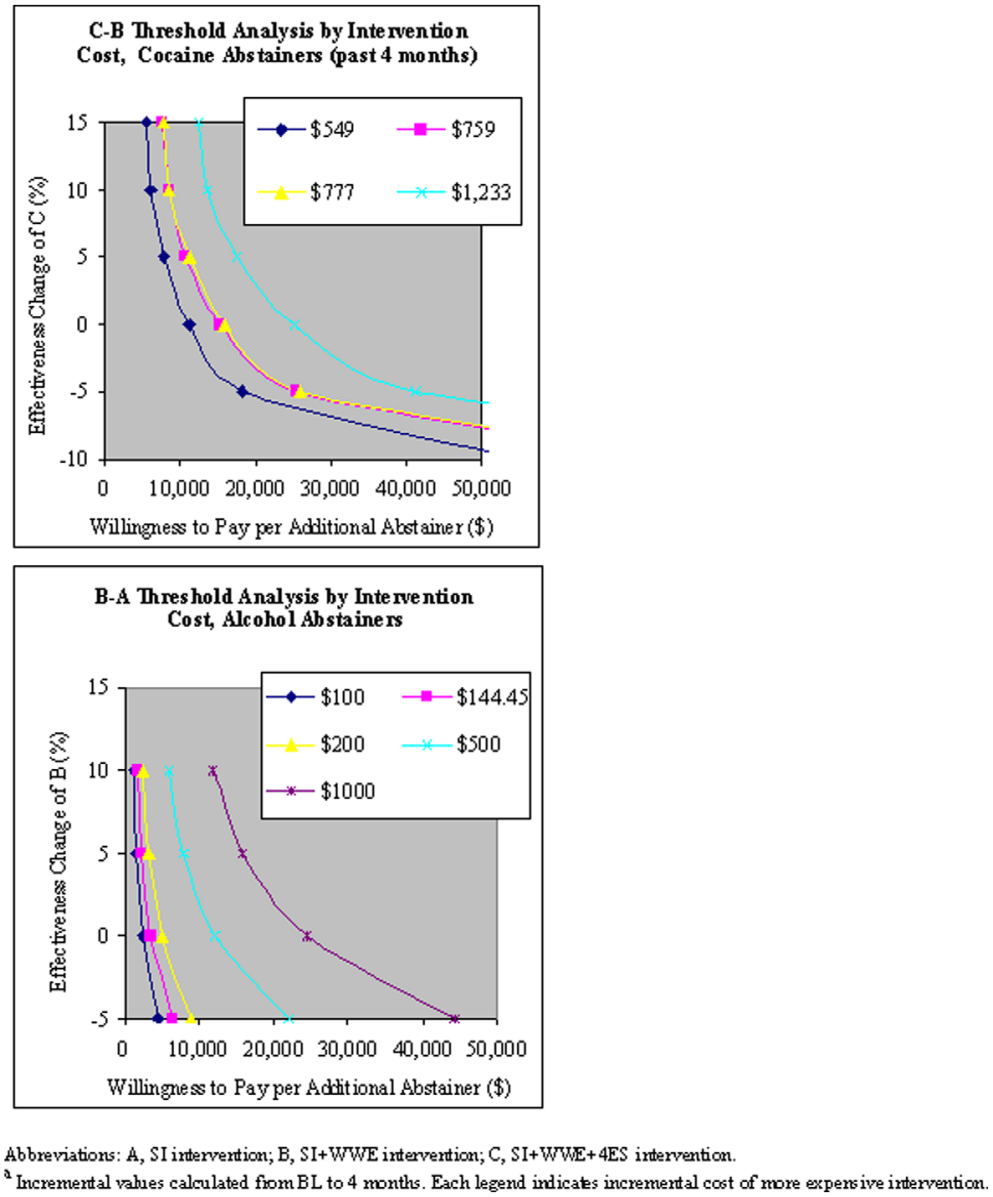
Willingness to Pay per Abstainer.

**Figure 2** portrays acceptability curves that address the uncertainty of the results, indicating the probability that WWE is cost-effective when compared to SI at a variety of willingness to pay (WTP) values. For example, the WWE intervention has a 0.5 probability of being cost-effective if society's WTP is $20,000 per additional cocaine abstainer (past 30 days) or $2,600 per additional alcohol abstainer. If the WTP is $25,000, it has a roughly 0.72 probability of being cost-effective for alcohol abstainers. **Figure 3** presents willingness to pay depending on intervention costs and changes in intervention effectiveness in achieving per additional cocaine abstainer and alcohol abstainer. For example, a 5% increase in effectiveness for 4ES (at a cost of $549) relative to WWE and for WWE (at a cost of $500) relative to SI yields a willingness to pay of less than $10,000 per additional cocaine abstainer and per additional alcohol abstainer, respectively.

## Discussion

To our knowledge, this is the first cost-effectiveness analysis simultaneously examining cocaine and alcohol abuse in women. The outcome of preventing heavy drinkers proved highly sensitive to changes in effectiveness values. Moreover, the magnitude of an ICER in the base case did not always predict its sensitivity. Decreasing the effectiveness of WWE for alcohol abstainers by 5% and varying the costs from $100 to $1,000 yielded significantly different ICER ranges at 4 M ($5,128–$51,282) and at 12 M ($40,000–$400,000), despite comparable base case ICERs ($3,611 at 4 M and $7,223 at 12 M). In cases of dominance among cocaine outcomes, the sensitivity to switching points varied considerably. It took only a small improvement (5%) in cocaine abstainers (past 30 days) at 12 M for WWE compared to SI to achieve a low ICER ($8,755); yet the same percentage improvement for 4ES failed to eliminate domination by WWE, and doubling that improvement led to a significantly higher ICER ($78,525). Although SI dominated WWE in the base case for cocaine free days, increasing effectiveness of WWE by 5% and varying incremental costs from $100 to $1,000 yielded low ICERs ranging from $94 to $936 at 4 M and $136 to $1,364 at 12 M.

Our results yielded ICERs that compare favorably to those in the literature, although one needs to be cautious in comparing results across different studies. CEAs alongside randomized controlled trials that examine the cost per obtaining an additional cocaine or alcohol abstainer, aside from analyses of contingency management (CM) programs, are scarce if not non-existent. We identified one study that examined costs and treatment readmission rates [35] and one that looked at costs per abstainer from substance abuse [36], both drawing on calculated ratios of the cost per probability of success. Other studies examined the incremental cost of extending drug abstinence. To extend the longest duration of cocaine and opioid abstinence by an additional week, Olmstead and Petry [15] report costs of $212 and $166 for voucher-based and prize-based CM interventions, respectively, compared to standard outpatient treatment. To reduce an additional day of drug use among out-of-treatment substance abusers at risk for HIV, Zarkin et al. [21] measured ICERs ranging from $36 to $140 for an enhanced intervention compared to SI. Schumacher et al. [37] estimated costs of two addiction interventions compared to usual care to be $1,007 and $1,244 for an additional week of drug abstinence among homeless persons. For non-abstinence outcomes, Zarkin et al. [13] determined that medical management combined with either naltrexone or naltrexone and acamprosate was cost-effective in avoiding heavy drinking (using the same heavy drinking definition as ours), with ICERs of $2,847 and $8,095. Jofre-Bonet et al. [38] examined the cost-effectiveness of disulfiram in addition to methadone maintenance for treating cocaine dependence, yielding an incremental cost of $73 per day of cocaine reduced.

The cost-effectiveness literature on substance abuse contains comparable analyses for treating heroin abuse, where: 7-day abstinence costs under AUD$4,000 (US$2,520) per additional patient for three detoxification methods compared to conventional outpatient treatment, the latter of which was also dominated by buprenorphine; [39] in prison, methadone treatment compared to no treatment costs AUD$38 (US$22) per additional heroin free day; [40] buprenorphine was dominated by methadone maintenance for obtaining an additional heroin free day [41]. The cost to prevent a recent quitter from smoking (relapse prevented) among low-income pregnant women was $1,217 [42].

As a benchmark of comparison for determining the worth of a QALY for alcohol treatment in the UK, two studies [14], [20] noted a range consistent with National Institute of Clinical Excellence (NICE) valuation of £20,000–30,000 ($37,768–56,652) per QALY gained. Additional studies reported the total benefits gained by society, determining the cost-effectiveness of: alcohol prevention [43] and a screening and brief intervention for excessive alcohol use in a primary care setting [16] in terms of QALYs; and acamprosate therapy in terms of cost savings per additional abstinent alcoholic [44].

We urge caution, however, not only in comparing our results to QALYs, but also in using QALYs as a measure for substance abuse prevention and treatment in an overarching social utility exercise (e.g. league table) because they overlook broader societal benefits and concerns [45]. As a single quantitative scale, QALYs assign less weight to the disabled and elderly in health care rationing and introduce problems of incommensurability [46], [47]. Moreover, since incremental CEAs yield ICERs that are reflective of their comparators, a low ICER may either indicate an ineffective and/or costly comparator or highly effective and/or inexpensive intervention. Thus, it is important, as we did in this study, to compare interventions to clinically relevant comparators. Economic considerations should follow clinical input in the decision making process [46].

The need for further research is clear. In 2003, the U.S. spent $21 billion (1.3% of total health care spending) on treatment for substance use disorders, $4.83 billion (23%) of which was spent by Medicare and Medicaid [48]. By comparison, Medicare costs for end stage renal disease (ESRD), which affected over half a million patients in 2007 [49], were an estimated $20.8 billion in 2007 and $18.1 billion in 2004 [50] – close to spending on treatment for substance abuse, which affects a much larger population (about 23.1 million in 2008) [1]. ESRD is very expensive to treat, and the relatively small group of ESRD patients deserve medically necessary and appropriate care; in light of the greater population affected by substance abuse and its broad societal costs ($180.9 billion for drug abuse in 2002) [4], however, it is sensible to direct additional attention and resources toward identifying and implementing effective and efficient substance abuse treatments. Our study has several limitations. First, generalizability is limited since the study was conducted in St. Louis and targeted drug use and high-risk sexual behaviors in women. Second, substance abuse prevention and treatment positively impact housing and employment sectors [51], costs and frequency of crime [52], [53], [45], and the development of the children of users [54], suggesting our results underestimated the full economic impact of treatment. Third, the intervention contained components designed for HIV prevention, rather than exclusively focusing on substance abuse, which reflects important interactions but may not be as targeted as needed for policy and treatment purposes. Fourth, the analysis did not include the incremental differences between 4 months and 12 months, though that information is derivable from data presented. Fifth, our study did not measure the longest duration of abstinence for either cocaine or alcohol. Sixth, the effectiveness outcomes were measured by self report, and biochemical verification was not available for the relevant endpoints used in our CEA.

## Supporting Information

Checklist S1 **CONSORT Checklist.**
(DOC)

Protocol S1 **Trial Protocol.**
(DOC)

Flow Diagram S1 **CONSORT Flow Diagram.**
(TIF)

Table S1 One-way Sensitivity Analyses: Costs.
(DOC)

Table S2 One-way Sensitivity Analyses: Outcomes.
(DOC)

Table S3 Two-way Sensitivity Analyses.
(DOC)

## References

[1] U.S. Department of Health and Human Services (2009). Results from the 2008 national survey on drug use and health: national findings.. http://www.oas.samhsa.gov/nsduh/2k8nsduh/2k8Results.pdf.

[2] U.S. Department of Health and Human Services (2011). Results from the 2010 national survey on drug use and health: summary of national findings.. http://www.oas.samhsa.gov/nsduh/2k10nsduh/2k10Results.pdf.

[3] Mokdad AH, Marks JS, Stroup DF, Gerberding JL (2004). Actual causes of death in the United States, 2000.. JAMA.

[4] Office of National Drug Control Policy (2004). The Economic Costs of Drug Abuse in the United States, 1992–2002.

[5] Rawson RA, Huber A, McCann M, Shoptaw S, Farabee D (2002). A comparison of contingency management and cognitive-behavioral approaches during methadone maintenance treatment for cocaine dependence.. Arch Gen Psychiatry.

[6] Penberthy JK, Ait-Daoud N, Vaughan M, Fanning T (2010). Review of treatment for cocaine dependence.. Curr Drug Abuse Rev.

[7] Anton RF, O'Malley SS, Ciraulo DA, Cisler RA, Couper D (2006). Combined pharmacotherapies and behavioral interventions for alcohol dependence: the COMBINE study: a randomized controlled trial.. JAMA.

[8] Cottler LB, Compton WM, Abdallah AB, Cunningham-Williams R, Abram F (1998). Peer-delivered interventions reduce HIV risk behaviors among out-of-treatment drug abusers.. Public Health Rep.

[9] National Institute on Drug Abuse (2008). Epidemiologic trends in drug abuse, vol. 1.

[10] National Institute on Drug Abuse (2011). Epidemiologic trends in drug abuse.

[11] Ruger JP, Abdallah AB, Leukens C, Cottler L (2010). Cost-effectiveness of interventions to prevent HIV and STDs among drug-using women: a randomized controlled trial.. http://ssrn.com/abstract=1619407.

[12] Ruger JP, Abdallah AB, Cottler L (2010). Costs of HIV prevention among out-of-treatment drug-using women: results of a randomized controlled trial.. Public Health Rep.

[13] Zarkin GA, Bray JW, Aldridge A, Mitra D, Mills MJ (2008). Cost and cost-effectiveness of the COMBINE study in alcohol-dependent patients.. Arch Gen Psychiatry.

[14] Drummond C, Coulton S, James D, Godfrey C, Parrott S (2009). Effectiveness and cost-effectiveness of a stepped care intervention for alcohol use disorders in primary care: pilot study.. Br J Psychiatry.

[15] Olmstead TA, Petry NM (2009). The cost-effectiveness of prize-based and voucher-based contingency management in a population of cocaine- or opioid-dependent outpatients.. Drug Alcohol Depend.

[16] Tariq L, van den Berg M, Hoogenveen RT, van Baal PHM (2009). Cost-effectiveness of an opportunistic screening programme and brief intervention for excessive alcohol use in primary care.. PLoS One.

[17] Olmstead TA, Sindelar JL, Petry NM (2007). Cost-effectiveness of prize-based incentives for stimulant abusers in outpatient psychosocial treatment programs.. Drug Alcohol Depend.

[18] Sindelar J, Elbel B, Petry NM (2007). What do we get for our money? Cost-effectiveness of adding contingency management.. Addiction.

[19] Sindelar JL, Olmstead TA, Peirce JM (2007). Cost-effectiveness of prize-based contingency management in methadone maintenance treatment programs.. Addiction.

[20] UKATT Research Team (2005). Cost effectiveness of treatment for alcohol problems: findings of the randomised UK alcohol treatment trial (UKATT).. BMJ.

[21] Zarkin GA, Lindrooth RC, Demiralp B, Wechsberg W (2001). The cost and cost-effectiveness of an enhanced intervention for people with substance abuse problems at risk for HIV.. Health Serv Res.

[22] Simpson DD, Joe GW, Fletcher BW, Hubbard RL, Anglin MD (1999). A national evaluation of treatment outcomes for cocaine dependence.. Arch Gen Psychiatry.

[23] Echeburua E, De Medina RB, Aizpiri J (2009). Personality disorders among alcohol-dependent patients manifesting or not manifesting cocaine abuse: a comparative pilot study.. Subst Use Misuse.

[24] Gold MR, Siegel JE, Russell LB, Weinstein MC (1996). Cost-effectiveness in health and medicine.

[25] Cunningham RM, Cottler LB, Compton WM (1996). Are we reaching and enrolling at-risk drug users for prevention studies?. J Drug Issues.

[26] Wechsberg WM, MacDonald BR, Dennis ML, Inciardi JA, Surratt HL (1997). The standard intervention for reduction in HIV risk behavior: protocol changes suggested by the continuing HIV/AIDS epidemic.

[27] Resch S, Altice FL, Paltiel AD (2005). Cost-effectiveness of HIV screening for incarcerated pregnant women.. J Acquir Immune Defic Syndr.

[28] Vocci FJ, Elkashef A (2005). Pharmacotherapy and other treatments for cocaine abuse and dependence.. Curr Opin Psychiatry.

[29] Dackis CA, Kampman KM, Lynch KG, Pettinati HM, O'Brien CP (2005). A double-blind, placebo-controlled trial of modafinil for cocaine dependence.. Neuropsychopharmacology.

[30] McKay JR, Alterman AI, Cacciola JS, Rutherford MJ, O'Brien CP (1997). Group counseling versus individualized relapse prevention aftercare following intensive outpatient treatment for cocaine dependence: initial results.. J Consult Clin Psychol.

[31] Gueorguieva R, Wu R, Pittman B, Cramer J, Rosenheck RA (2007). New insights into the efficacy of naltrexone based on trajectory-based reanalyses of two negative clinical trials.. Biol Psychiatry.

[32] Fenwick E, O'Brien BJ, Briggs A (2004). Cost-effectiveness acceptability curves – facts, fallacies and frequently asked questions.. Health Econ.

[33] Fenwick E, Claxton K, Sculpher M (2001). Representing uncertainty: the role of cost-effectiveness acceptability curves.. Health Econ.

[34] Lothgren M, Zethraeus N (2000). Definition, interpretation and calculation of cost-effectiveness acceptability curves.. Health Economics.

[35] Barnett PG, Swindle RW (1997). Cost-effectiveness of inpatient substance abuse treatment.. Health Serv Res.

[36] Weisner C, Mertens J, Parthasarathy S, Moore C, Hunkeler EM (2000). The outcome and cost of alcohol and drug treatment in an HMO: day hospital versus traditional outpatient regimens.. Health Serv Res.

[37] Schumacher JE, Mennemeyer ST, Milby JB, Wallace D, Nolan K (2002). Costs and effectiveness of substance abuse treatments for homeless persons.. J Ment Health Policy Econ.

[38] Jofre-Bonet M, Sindelar JL, Petrakis IL, Nich C, Frankforter T (2004). Cost effectiveness of disulfiram: treating cocaine use in methadone-maintained patients.. J Subst Abuse Treat.

[39] Shanahan MD, Doran CM, Digiusto E, Bell J, Lintzeris N (2006). A cost-effectiveness analysis of heroin detoxification methods in the Australian National Evaluation of Pharmacotherapies for Opioid Dependence (NEPOD).. Addict Behav.

[40] Warren E, Viney R, Shearer J, Shanahan M, Wodak A (2006). Value for money in drug treatment: economic evaluation of prison methadone.. Drug Alcohol Depend.

[41] Harris AH, Gospodarevskaya E, Ritter AJ (2005). A randomised trial of the cost effectiveness of buprenorphine as an alternative to methadone maintenance treatment for heroin dependence in a primary care setting.. Pharmacoeconomics.

[42] Ruger JP, Weinstein MC, Hammond SK, Kearney MH, Emmons KM (2008). Cost-effectiveness of motivational interviewing for smoking cessation and relapse prevention among low-income pregnant women: a randomized controlled trial.. Value Health.

[43] Månsdotter AM, Rydberg MK, Wallin E, Lindholm LA, Andréasson S (2007). A cost-effectiveness analysis of alcohol prevention targeting licensed premises.. Eur J Public Health.

[44] Schädlich PK, Brecht JG (1998). The cost effectiveness of acamprosate in the treatment of alcoholism in Germany. Economic evaluation of the Prevention of Relapse with Acamprosate in the Management of Alcoholism (PRAMA) Study.. Pharmacoeconomics.

[45] Sindelar JL, Jofre-Bonet M, French MT, McLellan AT (2004). Cost-effectiveness analysis of addiction treatment: paradoxes of multiple outcomes.. Drug Alcohol Depend.

[46] Ruger JP (2006). Health, capability, and justice: toward a new paradigm of health ethics, policy and law.. Cornell J Law Public Policy.

[47] Ruger JP (2009). Health and Social Justice.

[48] Mark TL, Levit KR, Coffey RM, McKusick DR, Harwood HJ (2007). National Expenditures for Mental Health Services and Substance Abuse Treatment, 1993–2003. SAMHSA Publication No. SMA 07-4227.

[49] Renalbusiness.com (2009). Many new ESRD patients haven't seen nephrologist.. http://www.renalbusiness.com/news/2009/09/many-new-esrd-patients-haven-t-seen-nephrologist.aspx.

[50] United States Renal Data System (2009). USRDS 2009 Annual Data Report: Atlas of Chronic Kidney Disease and End-Stage Renal Disease in the United States, vol. 3.

[51] Milby JB, Schumacher JE, Wallace D, Vuchinich R, Mennemeyer ST (2010). Effects of sustained abstinence among treated substance-abusing homeless persons on housing and employment.. Am J Public Health.

[52] Basu A, Paltiel AD, Pollack HA (2008). Social costs of robbery and the cost-effectiveness of substance abuse treatment.. Health Econ.

[53] Sindelar JL, Olmstead TA, Jones A (2006). Illicit drugs and drug-related crime.. The Elgar Companion to Health Economics.

[54] Ackerman JP, Riggins T, Black MM (2010). A review of the effects of prenatal cocaine exposure among school-aged children.. Pediatrics.

