# Correction: Cost-Effectiveness of Peer-Delivered Interventions for Cocaine and Alcohol Abuse among Women: A Randomized Controlled Trial

**DOI:** 10.1371/annotation/d9d8b547-8154-48a1-b527-d0c51285ed3c

**Published:** 2012-08-03

**Authors:** Jennifer Prah Ruger, Arbi Ben Abdallah, Craig Luekens, Linda Cottler

There is an error in the citation of the article regarding the first author's name. The correct citation is: Ruger JP, Abdallah AB, Luekens C, Cottler L (2012) Cost-Effectiveness of Peer-Delivered Interventions for Cocaine and Alcohol Abuse among Women: A Randomized Controlled Trial. PLoS ONE 7(3): e33594. doi:10.1371/journal.pone.0033594 

