# Supplementary material for: Cost-Effectiveness of Peer-Delivered Interventions for Cocaine and Alcohol Abuse among Women: A Randomized Controlled Trial
Source: PLoS One. 2012 Mar 20;7(3):e33594. doi: 10.1371/journal.pone.0033594 (PMC3308978; doi:10.1371/journal.pone.0033594)
Supplement: Protocol S1 — Trial Protocol. (DOC) [file pone.0033594.s002.doc]

| **ECONOMIC EVALUATION OF HIV-PREVENTION AMONG DRUG USING WOMEN** |
| --- |

**Objective: To evaluate the incremental cost-effectiveness of a three-arm public health intervention to prevent HIV and STDs in vulnerable, at-risk, drug-using women.**

In any given year, poor and minority women are disproportionately infected with HIV as compared to their male counterparts (SAMHSA, 2002). Women are primarily infected with HIV through injection drug use (48%) or heterosexual transmission from an infected partner (36%), who is also often a drug user (54.8%) (SAMHSA, 2002). Women can also become infected with HIV and other STDs through high-risk sexual behaviors associated with the use of other drugs, including crack/cocaine, and with the perceived need to exchange sex for drugs or money. This study of this K award proposes to evaluate the costs and incremental cost-effectiveness of three public health intervention arms: (i) a modified NIDA standard intervention (SI); (ii) the SI and well women exam (SI +WWE); and (iii) the SI and WWE plus four educational sessions (SI+WWE+4ES) to prevent HIV/STDs in at-risk, drug-using women. This study builds on a NIDA-funded project, the Women Teaching Women (WTW) study (DA11622, PI: Linda B. Cottler). The parent project assesses behavior change at 4 and 12 months post-intervention.

**Specific aims:**

1. to conduct a cost analysis of each of the three intervention arms;

2. to evaluate the incremental cost-effectiveness of the three interventions vis-à-vis each other;

3. to conduct an incremental cost-utility analysis determining the net cost per quality-adjusted life year (QALY) saved respectively by each intervention arm, vis-à-vis each other.

| **Background, Significance, and Rationale** |
| --- |

**HIV/STDs and Drug-Using Women**

HIV/AIDS rates for women and African Americans have been increasing as a result of drug use and sexual risk-taking (Karon & Rosenberg et al., 1996). The leading cause of death for African American women ages 25-44 is AIDS (CDC, 1997). Women older than 13 account for 15% of AIDS cases in the U.S., cases primarily due to shared needles or to heterosexual contact with drug users or others involved in high-risk behavior (CDC, 1997). In some cities, the proportion of AIDS cases attributed to heterosexual IDUs has exceeded the proportion attributed to non-IDU homo-sexual men. African Americans account for nearly 13% of the U.S. population and 57% of new HIV infections.

**Gender Specific Intervention Strategies for Out-Of-Treatment Drug-Using Women**

Research suggests that interventions delivered by peers and tailored to a woman’s individual sexual and drug behaviors have the greatest likelihood of increasing her perceptions of the seriousness of HIV/AIDS. The Health Belief Model (HBM) is thought to be effective in health behavior change among IDUs (Janz & Becker, 1984; Rosentstock, 1974). Specifically, perceived self-efficacy and susceptibility have been found to be related to safer injection practices, particularly among African American IDUs (Falck & Siegal et al.,1995). It is also thought that for interventions to have the greatest impact, individuals must be actively engaged in message delivery as in one-on-one and small peer group communication (Rugg, 1990).

**Cost-Effectiveness Studies of HIV Prevention Programs**

In general, the literature has demonstrated that most interventions to prevent sexual transmission of HIV in the U.S. are cost-effective or even cost-saving (from a societal perspective, a cost-saving program saves society money in the long run, by preventing large expenditures in HIV-related medical care). Interventions focused on homosexual and bisexual men, for example, have been found to be cost-saving in a number of different settings and with different intervention types (Holtgrave & Kelly, 1997; Kahn & Haynes-Sanstad, 1997; Pinkerton, Holtgrave, & Valdiserri et al., 1997; Pinkerton & Holtgrave, 1998a, 1998b; Tao & Remafedi, 1998). In addition, a program delivering a small-group cognitive-behavioral intervention to women attending urban health care clinics was found to be cost-effective (Holtgrave & Kelly, 1996), while a similar intervention focused on women with severe mental illness was not deemed cost-effective (Pinkerton, Johnson-Masotti, & Otto-Salaj et al., 2001). Another program involving small-group sessions and peer leader training focused on women and men with severe mental illness varied in its cost-effectiveness by gender and type of intervention (Johnson-Masotti, & Pinkerton et al., 2000).

It is difficult to compare the results of different economic evaluations due to the diversity of evaluation types and methods used (e.g., cost-effectiveness analysis, cost-utility analysis, and incremental cost-utility analysis; different measures of effectiveness; different mathematical modeling techniques) and to determine which of the different program characteristics (e.g., targeted populations & intervention types) lead one program to appear more cost-effective than others. Still, studies are suggestive of some overall trends. In particular, programs employing small-group, safer sex skills building, cognitive-behavioral sessions or peer leader community-level norm change foci as intervention techniques typically emerge as particularly cost-effective intervention modalities, as do programs aimed at high-risk groups (Holtgrave & Pinkerton, 1998; Pinkerton, Johnson-Masotti, & Holtgrave et al., 2001). For example, a program for gay and bisexual male youth and young adults (Remafedi, 1994) that featured risk reduction counseling, peer education, optional HIV testing and counseling, and referral to medical and psycho-social services, was found to be moderately cost-effective (Tao & Remafedi, 1998). However, because this program was tailored to the specific needs of young men who have sex with men, it is of limited relevance to programs targeted specifically for minority populations, youth, or at-risk women. There appears to be a gap in the cost-effectiveness literature with regard to gender-specific interventions focused on at-risk, drug-abusing women*.*

| **Preliminary Studies and Results** |
| --- |

Recruitment, Randomization and Intervention Delivery for Drug-Using Women

| Table 3: Recruitment and Intervention Delivery (as of 5/10/02)  Intervention Arm Randomized Exam Completed | | |
| --- | --- | --- |
| Group A (SI) | 67 | N/A |
| Group B (SI +WWE) | 80 | 56 |
| Group C (SI+WWE+4ES) | 74 | 54 |

The WTW study is now in its second of five years and records are being kept on randomization and intervention delivery. It is still in the early stages of the project, but Table 3 outlines participant numbers (as of 5/10/02) for the three-arm intervention.

**Power Calculations**

WTW study investigators proposed a randomized controlled study with an equal number of subjects in each of the three prevention groups (Groups A, B, and C). It was estimated that a total sample of approximately 650 women (who completed the 4 month and 12 month post-intervention follow-ups) would be adequate to determine whether there are differential effects across Groups. The targeted number of participants per group is approximately 215. Table 4 delineates differences in key outcome measures for a 2-sided test with a = 0.05 and with a power of 80% in the direction of improvement (decrease in the level of risky behavior).

| **Table 4: Computed Critical Effect Size for WTW Study** | |
| --- | --- |
| **Relevant Measure** | **Difference** |
| Number of times used crack cocaine, past 30 days | 10.3 |
| Number of days woman had sex, past 30 days | 1.7 |
| Number of sex partners, past 30 days | 0.4 |
| Injection drug use, past 30 days | 5% |
| Number of times had unprotected sex, past 30 days | 1.7 |

| **Research Design and Methods** |
| --- |

**Study Design, Sample, and Public Health Interventions**

The WTW study is an HIV and STD prevention project focused on out-of-treatment IDUs and crack cocaine users (DA11622, PI: Linda Cottler). The prevention model is peer-delivered and tailored specifically to women’s needs. The intervention is designed to bring the HIV prevention message to women in a public health environment. The three-arm intervention assesses the differential impact of: (i) a modified NIDA Cooperative Agreement Standard Intervention (SI); (ii) the SI + Well-Woman Exam (SI+WWE); and (iii) the SI+Well-Woman Exam + 4 Educational Sessions (SI+WWE+4ES). The SI is delivered by peers; the Well-Woman Examination is conducted by a nurse practitioner; the four 2 hour educational sessions are conducted by peer facilitators from area drug treatment programs paired with a community mental health or health professional. Women have been randomly assigned to one of three peer-delivered interventions.

**Cost, Cost-Effectiveness, and Cost-Utility Analyses**

**Cost Analyses**

I will work with colleagues on the parent study to conduct a cost analysis of each of the three intervention arms from both a community-based organization perspective and from a more comprehensive “societal” perspective (Drummond & Stoddart et al., 1987; Gold & Siegel et al., 1996). Standard techniques for conducting cost analyses of public health/prevention programs are described in the literature (Gorsky, 1996; Gorsky & Teutsch, 1995), and have been applied by a number of researchers to evaluate HIV prevention interventions (Johnson-Masotti & Pinkerton et al., 2000; Pinkerton, Holtgrave, & DiFranceisco et al., 1998; Pinkerton & Holtgrave et al., 2000; Pinkerton, Holtgrave, & Valdiserri et al., 1997). The costs assessed in this study are consistent with those techniques (Gold & Siegel et al., 1996). The costs are now being collected in the parent study.

Building on previous research in this area, and taking into consideration the format of the WTW Programs, the main cost categories include personnel costs for peer facilitators, nurse practitioners, community health professionals, and trainers (e.g., salary or hourly wage, plus fringe benefits); program space and facility costs (rent, insurance, equipment and utilities); administrative personnel costs, materials (e.g., brochures and exam materials); monetary incentives for participants; durable equipment (e.g., filing cabinets and computers) and office supplies (e.g., paper and forms). Set-up costs (e.g., costs incurred at the point of program implementation) will also be assessed. These include training costs (e.g., for personnel and program staff) and training materials. The costs of materials used in the intervention, training, remuneration and other items will be determined from billing records and expense reports maintained by the research team. (Only costs related to the implementation of the intervention will be included; study recruitment, development of the survey instrument, and other activities associated with the research objectives of the study will be excluded). Time utilization diaries are being used to track facilitators’ time spent in training, preparation, provision of group and individual sessions, and other activities. Diary, salary, and fringe benefit information will be combined to determine overall personnel costs. Space utilized for interventions will be assessed as a portion of the overall square footage available in the facility, taking into account the alternative uses for that space. This will be combined with room rental, utility (electricity, heating, telephone), and insurance cost information to determine the facilities cost.

To estimate opportunity costs, participants are asked questions about the travel and transportation to and from the intervention site and whether they required childcare to attend. Transportation costs will also be assessed from administrative records, where transportation is provided by the program. In general, the best approximation of the opportunity cost of time for drug-abusing women is to base the wage rate on women with the same employment record and educational status in the labor force (Gold & Siegel et al., 1996).

**Cost-Effectiveness Analysis**

I will combine the cost information obtained through the cost analysis with self-reported behavioral change data to evaluate the cost-effectiveness of the WTW interventions. The main steps in performing the cost-effectiveness analysis include: (i) estimation of all intervention-related costs (cost analysis); (ii) mathematical modeling to translate reported changes in intervention participants’ sexual behaviors into an estimate of the number of HIV infections averted by the intervention; (iii) computation of relevant cost-effectiveness ratios; and (iv) conducting sensitivity analyses to examine the robustness of key parameters by varying their values, singly and in combination, within a plausible range (Gold & Siegel et al., 1996). A simulation approach will be used to estimate the distribution of the cost-effectiveness ratios (Mullahy & Manning, 1994; Gold & Siegel et al., 1996).

Standardized methodologies have been developed for conducting such analyses (Pinkerton & Holtgrave, 1998a,b; Weinstein & Graham et al., 1989) that use mathematical models of HIV transmission to translate reported changes in sexual behavior into an estimate of the number of HIV infections averted by the intervention (Pinkerton & Abramson, 1993, 1998). I will use established baseline parameters (e.g., estimates of lifetime costs of treating HIV, the number of QALYs lost when one becomes infected (Holtgrave & Pinkerton, 1997), and the effectiveness of condoms in preventing HIV transmission (Pinkerton & Abramson, 1997)) to conduct CEA and CUA analyses. I will extend the reach of the parent study by performing the CEA and CUA analyses described herein (that were not a part of the parent study).

I will use a Bernoullian mathematical model of HIV transmission (Pinkerton & Abramson, 1993, 1998) to translate participants’ self-reported sexual behaviors into estimates of their risk of contracting or transmitting HIV as a consequence of those behaviors. Changes in these risk estimates will be converted into an estimate of the number of HIV infections averted by the intervention, as described in detail elsewhere (Pinkerton & Holtgrave, 1998 a,b; Pinkerton, Holtgrave, & Leviton et al., 1998; Weinstein & Graham et al., 1989). The number of infections averted by an intervention is a function of the number of intervention participants; the number of acts of unprotected and condom-protected vaginal and anal receptive intercourse reported by participants at baseline and each follow-up, and the number of partners for each of these sexual behaviors; the prevalence of HIV among intervention participants and their sex partners; the per-act transmission probabilities of unprotected vaginal and anal receptive intercourse; and the effectiveness of condoms at preventing HIV transmission. For example, the probability of a young woman, who only has sex with men, becoming infected as a result of n_1_ acts of unprotected receptive anal intercourse, n_2_ acts of condom-protected receptive anal intercourse, n_3_ acts of unprotected receptive vaginal intercourse, and n_4_ acts of protected receptive vaginal intercourse with each of m partners is approximately,

P = 1 – { (1 – π) + π (1 – p_1_)^n1^ (1 – p_2_)^n2^ (1 – p_3_)^n3^ (1 – p_4_)^n4^ }^m^

where π is the estimated prevalence of HIV infection among her partners and p_k_ is the relevant per-act transmission probability (Pinkerton & Abramson, 1998; Weinstein & Graham et al., 1989), taking into account the imperfectly protective effect of latex condoms (Pinkerton & Abramson, 1997). Estimates of the per-act transmission probabilities can be found in the literature. In the base-case (main) analysis, I will use the following per-act transmission probabilities: 0.001 for receptive vaginal intercourse; and 0.02 for receptive anal intercourse (Katz & Gerberding, 1997; Mastro & de Vincenzi, 1996; Royce & Seña et al., 1997). I will vary these values in sensitivity analyses to see how the results change as a function of these parameter values. Condom effectiveness will be set at 90% in the base-case (Pinkerton & Abramson, 1997), and varied from 65% to 100% in sensitivity analyses. I will conduct a literature review to estimate the prevalence of HIV among partners of intervention participants. I will establish a base-case prevalence estimate based on the review and discussions with local public health officials, and I will conduct sensitivity analyses to examine the impact of different prevalence rates on the results of the main analysis.

I will compute several distinct cost-effectiveness ratios (cost per HIV infection averted). First, I will compute an “average” cost-effectiveness ratio for each intervention. The average cost-effectiveness ratio equals C/A, where C is the total cost of the prevention intervention and A is the number of infections it prevented (A=P(P_0_ – P_12_)), where P_0_ and P_12_ represent respectively the participant’s risk of becoming infected at baseline and 12 months follow-up. The average cost-effectiveness ratio is a measure of the economic efficiency of the intervention compared to a “do nothing” program. If, as I anticipate, for example, the SI+WWE+4ES is significantly more effective than the SI +WWE intervention, then a more appropriate comparison is provided by the “incremental cost-effectiveness ratio,” defined, for example, as: (C_SI+WWE+4ES_ – C_SI+WWE_) / (A_SI+WWE+4ES_ – A_SI+WWE_), where C_SI+WWE+4ES_  and C_SI+WWE_ are the respective costs of the two interventions, A_SI+WWE+4ES_ and A_SI+WWE_ are the respective numbers of averted infections. Thus, the incremental cost-effectiveness ratio is the ratio of the difference in costs between the two interventions and the difference in their effectiveness. These analyses will help determine whether the added cost of the (presumably) more effective SI+WWE+4ES intervention is justified by its greater health impact.

**Cost-Utility Analysis**

As recommended by *Cost-Effectiveness in Health and Medicine* (Gold & Siegel et al., 1996), I will conduct a cost-utility analysis, in which the outcome of interest is the net cost per quality-adjusted life year (QALY) saved by the intervention (Gorsky, 1996; Weinstein & Graham et al., 1989). For HIV prevention programs, the “average” cost-utility ratio equals (C – AT)/AQ, where C is the cost of the program, A is the number of infections averted by the program, T is the lifetime cost of treating a single case of HIV disease, and Q is the number of QALYs saved by preventing someone from becoming infected with HIV (Pinkerton & Holtgrave, 1998a,b). The net cost of an HIV prevention intervention equals the cost of the intervention minus savings in averted HIV/AIDS medical treatment costs. Thus, the net cost of an HIV prevention intervention that prevents A infections is C – AT. Similarly, the total number of QALYs saved by the intervention equals AQ. Health-related programs with cost-utility ratios less than $40,000 to $60,000 per QALY saved are generally considered cost-effective (Kaplan & Bush, 1982; Paltiel & Stinnett, 1998).

Standardized values of T and Q can be obtained from the literature (Holtgrave & Pinkerton, 1997) and a number of studies have used these estimates, facilitating comparisons. Estimates indicate that the lifetime cost of treating HIV infection is $195,188 (1996 dollars), and the number of QALYs lost by age 65 of someone who becomes infected at age 20 is 13.00, where both costs and QALYs are discounted into the future at 3% per year (Holtgrave & Pinkerton, 1997; Pinkerton & Holtgrave, 1998a; Pinkerton & Holtgrave et al., 2000). I will also independently calculate the number of lost QALYs for each participant, based on her age at the time of the intervention trial and based on published estimates of the distribution of QALYs lost due to HIV Infection (Pinkerton & Holtgrave, 1998). I will review the literature to update the cost and QALY estimates, if possible, for recent advances (e.g., lifetime HIV costs have also been estimated at $140,000, Holtgrave, 1998). I will conduct a sensitivity analysis to examine the impact of different values of lifetime HIV costs on the results of the main analysis. In addition to calculating the “average” cost-utility ratio for each intervention condition, as described above, I will also calculate an “incremental” ratio, for example: [ (C _SI+WWE+4ES_ – C _SI+WWE_) – (A _SI+WWE+4ES_ – A _SI+WWE_)T ] / (A _SI+WWE+4ES_ – A _SI+WWE_)Q, to directly compare the costs and outcomes of the three interventions, respectively. This ratio (the incremental cost per incremental QALY saved) will be compared with the $40,000 to $60,000 threshold to determine whether the (presumably) greater QALY savings associated with the SI+WWE+4ES intervention relative to the SI+WWE intervention, and the SI+WWE intervention relative to the SI intervention, are worth the (presumably) extra cost.

| **References** |
| --- |

Centers for Disease Control (CDC). (1997). Update: Trends in AIDS incidence, deaths, and prevalence-United States, 1996. *Morbidity and Mortality Weekly Report, 46* (No. SS-8).

Centers for Disease Control (CDC). (1998). Youth risk behavior surveillance-United States, 1997.

*Morbidity and Mortality Weekly Report, 47* (No. SS-3).

Drummond, M. F., Stoddart, G. L, & Torrance, G. W. (1987). *Methods for the Economic Evaluation of Health Care Programmes.* Oxford: Oxford University Press.

Falck, R. S., Siegal H. A., Wang, J., & Carlson, R. G. (1995). Usefulness of the health belief model in predicting HIV needle risk practices among injection drug users. *AIDS Education and Prevention, 7* (6), 523-533.

Gold, M. R., Siegel, J. E., Russell, L. B., & Weinstein, M. C. (Eds.). (1996). *Cost-effectiveness in health and medicine*. New York: Oxford University Press.

Gorsky, R. D. (1996). Cost of an intervention. In A. C. Haddix, S. M. Teutsch, P. A. Shaffer, & D. O.

Duñet (Eds.), *Prevention Effectiveness: A Guide to Decision Analysis and Economic Evaluation*. New York: Oxford University Press.

Gorsky, R. D., & Teutsch, S. M. (1995). Assessing the effectiveness of disease and injury prevention programs: Costs and consequences. *Morbidity and Mortality Weekly Report, RR-10,* 1-10.

Holtgrave, D. R. (Ed.). (1998). *Handbook of Economic Evaluation of HIV Prevention Programs.* New York: Plenum Press.

Holtgrave, D. R., & Kelly, J. A. (1996). Preventing HIV/AIDS among high-risk urban women: The cost-effectiveness of a behavioral group intervention. *American Journal of Public Health, 86,* 1442-1445.

Holtgrave, D. R., & Kelly, J. A. (1997). Cost-effectiveness of an HIV/AIDS prevention intervention for gay men. *AIDS and Behavior, 1,* 173-180.

Holtgrave, D. R., & Pinkerton, S. D. (1997). Updates of cost of illness and quality of life estimates for use in economic evaluations of HIV prevention programs. *Journal of Acquired Immune Deficiency Syndromes, 16*, 54-62.

Holtgrave, D. R., & Pinkerton, S. D. (1998). The cost-effectiveness of small group and community-level interventions. In D. R. Holtgrave (Ed.), *Handbook of Economic Evaluation of HIV Prevention Programs* (pp. 119-126). New York: Plenum Press.

Janz, N. K., & Becker, M. H. (1984). The Health Belief Model: A decade later. *Health Education*

*Quarterly, 11* (1), 1-47.

Johnson-Masotti, A. P., Pinkerton, S. D., Kelly, J. A., & Stevenson, L. Y. (2000). Cost-effectiveness of an HIV risk reduction intervention for adults with severe mental illness. *AIDS Care, 12*, 321-332.

Kahn, J. G., & Haynes-Sanstad, K. C. (1997). The role of cost-effectiveness analysis in assessing HIV-prevention interventions. AIDS and Public Policy Journal, 12, 21-30.

Kaplan, R. M., & Bush, J. W. (1982). Health-related quality of life measurement for evaluation research and policy analysis. *Health Psychology, 1*, 61-80.

Karon, J. M., Rosenberg, P. S., McQuillan, G., Gwinn, M., & Petersen, L. R. (1996). Prevalence of HIV infection in the United States, 1984 to 1992. *Journal of the American Medical Association, 276*, 126-131.

Katz, M. H., & Gerberding, J. L. (1997). Postexposure treatment of people exposed to the human

immunodeficiency virus through sexual contact or injection-drug use. *New England Journal of Medicine, 336,* 1098-1100.

Mastro, T. D., & de Vincenzi, I. (1996). Probabilities of sexual HIV-1 transmission. *AIDS, 10* (Suppl. A), S75-S82.

Mullahy, J., & Manning, W. G. (1994). Statistical issues in cost-effectiveness analyses. In F. Sloan (Ed.), *Valuing Health Care: Costs, Benefits and Effectiveness of Pharmaceuticals and Other Medical Technologies*. New York: Cambridge University Press.

Paltiel, A. D., & Stinnett, A. A. (1998). Resource allocation and the funding of HIV prevention. In D. R. Holtgrave (Ed.), *Handbook of Economic Evaluation of HIV Prevention Programs* (pp. 135-152). New York: Plenum Press.

Pinkerton, S. D., & Abramson, P. R. (1993). Evaluating the risks: A Bernoulli process model of HIV infection and risk reduction. *Evaluation Review, 17,* 504-528.

Pinkerton, S. D., & Abramson, P. R. (1997). Effectiveness of condoms in preventing HIV transmission. *Social Science and Medicine, 44,* 1303-1312.

Pinkerton, S. D., & Abramson, P. R. (1998). The Bernoulli-process model of HIV transmission:

Applications and implications. In D. R. Holtgrave (Ed.), *Handbook of Economic Evaluation of HIV Prevention Programs* (pp. 13-33). New York: Plenum Press.

Pinkerton, S. D., & Holtgrave, D. R. (1998a). A method for evaluating the economic efficiency of HIV behavioral risk reduction interventions. *AIDS and Behavior, 2*, 189-201.

Pinkerton, S. D., & Holtgrave, D. R. (1998b). Assessing the cost-effectiveness of HIV prevention

interventions: A primer. In D. R. Holtgrave (Ed.), *Handbook of economic evaluation of HIV prevention programs*. New York: Plenum Press.

Pinkerton, S. D., Holtgrave, D. R., DiFranceisco, W. J., Stevenson, L. Y., & Kelly, J.A. (1998). Cost-effectiveness of a community-level HIV risk reduction intervention. *American Journal of Public Health, 88*, 1239-1242.

Pinkerton, S. D., Holtgrave, D. R., & Jemmott, J. B. (2000). Economic analysis of an HIV risk reduction intervention for male African American adolescents. *Journal of Acquired Immune Deficiency Syndromes, 25*, 164-172.

Pinkerton, S. D., Holtgrave, D. R., Leviton, L. C., Wagstaff, D. A., & Abramson, P. R. (1998). Model-based evaluation of HIV prevention interventions. *Evaluation Review, 22*, 155-174.

Pinkerton, S. D., Holtgrave, D. R., & Valdiserri, R. O. (1997). Cost-effectiveness of HIV prevention skills training for men who have sex with men. *AIDS, 11*, 347-357.

Pinkerton, S. D., Johnson-Masotti, A. P., Holtgrave, D. R., & Farnham, P. G. (2001). Using cost

effectiveness league tables to compare interventions to prevent sexual transmission of HIV. *AIDS, 15*, 917-928.

Pinkerton, S. D., Johnson-Masotti, A. P., Otto-Salaj, L. L., Stevenson, L. Y., & Hoffmann, R. G. (2001). Cost-effectiveness of an HIV risk reduction intervention for mentally ill adults. *Mental Health Services Research, 3* (1), 45-55.

Rosentstock, I. M. (1974). Historical origins of the health belief model. *Health Education Monograph, 2,* 232.

Royce, R. A., Seña, A., Cates, W. Jr., & Cohen, M. S. (1997). Sexual transmission of HIV. *New England Journal of Medicine,* *336*, 1072-1078.

The Substance Abuse and Mental Health Services Administration (SAMHSA). (1996). *National Household Survey on Drug Abuse*. Retrieved from http://www.samhsa.gov/oas/nhsda/PE1996/HTTOC.HTM

Tao, G., & Remafedi, G. (1998). Economic evaluation of an HIV prevention intervention for gay and bisexual male adolescents. *Journal of Acquired Immune Deficiency Syndromes Human Retrovirology, 17,* 83-90.

Weinstein, M. C., Graham, J. D., Siegel, J. E., & Fineberg, H. V. (1989). Cost-effectiveness analysis of AIDS prevention programs: Concepts, complications, and illustrations. In C. F. Turner, H. G. Miller, & L. E. Moses (Eds.), *AIDS: Sexual Behavior and Intravenous Drug Use* (pp. 471-499). Washington, D.C.: National Academy Press.
