# Supplementary figures and images for: Cost-Effectiveness of Peer-Delivered Interventions for Cocaine and Alcohol Abuse among Women: A Randomized Controlled Trial
Source: PLoS One. 2012 Mar 20;7(3):e33594. doi: 10.1371/journal.pone.0033594 (PMC3308978; doi:10.1371/journal.pone.0033594)

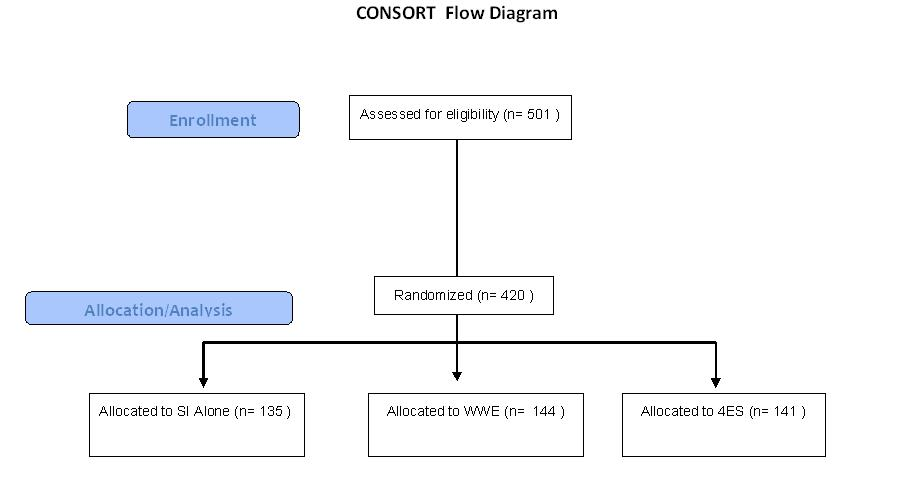

Supplement: Flow Diagram S1 — CONSORT Flow Diagram. (TIF) [file pone.0033594.s003.tif]
