# Supplementary material for: Cost-Effectiveness of Peer-Delivered Interventions for Cocaine and Alcohol Abuse among Women: A Randomized Controlled Trial
Source: PLoS One. 2012 Mar 20;7(3):e33594. doi: 10.1371/journal.pone.0033594 (PMC3308978; doi:10.1371/journal.pone.0033594)
Supplement: Table S1 — One-way Sensitivity Analyses: Costs. (DOC) [file pone.0033594.s004.doc]

|  | | | | | | **Table S1: One-Way Sensitivity Analyses: Costs^a^**   |  | | | | | | | | | | | | | | |  | | | | | | --- | --- | --- | --- | --- | --- | --- | --- | --- | --- | --- | --- | --- | --- | --- | --- | --- | --- | --- | --- | |  |  |  |  | |  |  | |  | |  |  |  |  |  | |  |  |  |  | | **Cocaine Outcomes** | |  |  |  | | |  | | **ICER (∆C/∆E, $), BL to 4mo** | | | | | | | | | | | |  |  | Cost per Occasion Averted^b^ | | |  | Cost per Episode Reduced^c^ | | | |  | Cost per Cocaine Free Day | |  | Cost per Abstainer, past 30 days | | |  | Cost per Abstai | | | Incremental Cost, B - Aer, past 4mo | Incremental C | st, C - B  WWE | 4ES | |  | WWE | | 4ES | |  | WWE | 4ES |  | WWE | | 4ES |  | WWE | 4ES | | ($144.45) | ($942.3) | (D) | ($146) | |  | ($498) | | ($3,490) | |  | (D) | (D) |  | ($14,445) | | ($18,846) |  | ($7,223) | ($18,846) | | $100 | $549^d^ | D | $85 | |  | $345 | | $2,033 | |  | D | D |  | $10,000 | | $10,980 |  | $5,000 | $10,980 | | $200 | $759^e^ | D | $118 | |  | $690 | | $2,811 | |  | D | D |  | $20,000 | | $15,180 |  | $10,000 | $15,180 | | $500 | $777^f^ | D | $121 | |  | $1,724 | | $2,878 | |  | D | D |  | $50,000 | | $15,540 |  | $25,000 | $15,540 | | $1,000 | $1,233^g^ | D | $191 | |  | $3,448 | | $4,567 | |  | D | D |  | $100,000 | | $24,660 |  | $50,000 | $24,660 | |  |  |  |  | |  |  | |  | |  |  |  |  |  | |  |  |  |  | |  |  |  |  |  | | |  | | **ICER (∆C/∆E, $), BL to 12mo** | | | | | | | | | | | |  |  | Cost per Occasion Averted^b^ | | |  | Cost per Episode Reduced^c^ | | | |  | Cost per Cocaine Free Day | |  | Cost per Abstainer, past 30 days | | |  | Cost per Abstainer, past 4mo | | | Incremental Cost, B - A | Incremental Cost, C - B | WWE | 4ES | |  | WWE | | 4ES | |  | W | E  4ES |  | WWE | | 4ES |  | WWE | 4ES | | ($144.45) | ($942.3) | (ED) | ($43) | |  | (D) | | ($1,059) | |  | (D) | ($504) |  | (D) | | (D) |  | ($3,611) | (D) | | $100 | $549^d^ | $233 | $25 | |  | D | | $617 | |  | D | $294 |  | D | | D |  | $2,500 | D | | $200 | $759^e^ | $465 | $35 | |  | D | | $853 | |  | D | $406 |  | D | | D |  | $5,000 | D | | $500 | $777^f^ | $1,163 | $36 | |  | D | | $873 | |  | D | $416 |  | D | | D |  | $12,500 | D | | $1,000 | $1,233^g^ | $2,326 | $56 | |  | D | | $1,385 | |  | D | $659 |  | D | | D |  | $25,000 | D | |  |  |  |  | |  |  | |  | |  |  |  |  |  | |  |  |  |  | | | | | | | | | | | | | | | | |  | | | | | | | | | | | | | | | | | | | |
| --- | --- | --- | --- | --- | --- | --- | --- | --- | --- | --- | --- | --- | --- | --- | --- | --- | --- | --- | --- | --- | --- | --- | --- | --- | --- | --- | --- | --- | --- | --- | --- | --- | --- | --- | --- | --- | --- | --- | --- | --- | --- | --- | --- | --- | --- | --- | --- | --- | --- | --- | --- | --- | --- | --- | --- | --- | --- | --- | --- | --- | --- | --- | --- | --- | --- | --- | --- | --- | --- | --- | --- | --- | --- | --- | --- | --- | --- | --- | --- | --- | --- | --- | --- | --- | --- | --- | --- | --- | --- | --- | --- | --- | --- | --- | --- | --- | --- | --- | --- | --- | --- | --- | --- | --- | --- | --- | --- | --- | --- | --- | --- | --- | --- | --- | --- | --- | --- | --- | --- | --- | --- | --- | --- | --- | --- | --- | --- | --- | --- | --- | --- | --- | --- | --- | --- | --- | --- | --- | --- | --- | --- | --- | --- | --- | --- | --- | --- | --- | --- | --- | --- | --- | --- | --- | --- | --- | --- | --- | --- | --- | --- | --- | --- | --- | --- | --- | --- | --- | --- | --- | --- | --- | --- | --- | --- | --- | --- | --- | --- | --- | --- | --- | --- | --- | --- | --- | --- | --- | --- | --- | --- | --- | --- | --- | --- | --- | --- | --- | --- | --- | --- | --- | --- | --- | --- | --- | --- | --- | --- | --- | --- | --- | --- | --- | --- | --- | --- | --- | --- | --- | --- | --- | --- | --- | --- | --- | --- | --- | --- | --- | --- | --- | --- | --- | --- | --- | --- | --- | --- | --- | --- | --- | --- | --- | --- | --- | --- | --- | --- | --- | --- | --- | --- | --- | --- | --- | --- | --- | --- | --- | --- | --- | --- | --- | --- | --- | --- | --- | --- | --- | --- | --- | --- | --- | --- | --- | --- | --- | --- | --- | --- | --- | --- | --- | --- | --- | --- | --- | --- | --- | --- | --- | --- | --- | --- | --- | --- | --- | --- | --- | --- | --- | --- | --- | --- | --- | --- | --- | --- | --- | --- | --- | --- | --- | --- | --- | --- | --- | --- | --- | --- | --- | --- | --- | --- | --- | --- | --- | --- | --- | --- | --- | --- | --- | --- | --- | --- | --- | --- | --- | --- | --- | --- | --- | --- | --- | --- | --- | --- | --- | --- | --- | --- | --- | --- | --- | --- | --- | --- | --- | --- | --- | --- | --- | --- | --- | --- | --- | --- | --- | --- | --- | --- | --- | --- | --- | --- | --- | --- | --- | --- | --- | --- | --- | --- | --- | --- | --- | --- | --- | --- | --- | --- | --- | --- | --- | --- | --- | --- | --- | --- | --- | --- | --- | --- | --- | --- | --- | --- | --- | --- | --- | --- | --- | --- | --- | --- | --- | --- | --- | --- | --- | --- | --- | --- | --- | --- | --- | --- | --- | --- | --- | --- | --- | --- | --- | --- | --- | --- | --- | --- |
|  |  | | | | | | | | | | | | | | | | | | | | | | | | | | | | | | | | | | | | | | | | |
|  |  | | | | | | | | | | | | | | | | | | | | | | | | | | | | | | | | | | | | | | | | |
|  | | | | | |  | | | | | | | | | | | | | | | |  | | | | | | | | | | | | | | | | | | | |
|  | | | | | |  | | | | | | | | | | | | | | | |  | | | | | | | | | | | | | | | | | | | |
|  | | | | | |  | | | | | | | | | | | | | | | |  | | | | | | | | | | | | | | | | | | | |
|  | | | | | |  | | | | | | | | | | | | | | | |  | | | | | | | | | | | | | | | | | | | |
|  | | | | | |  | | | | | | | | | | | | | | | |  | | | | | | | | | | | | | | | | | | | |
|  | | | | | |  | | | | | | | | | | | | | | | |  | | | | | | | | | | | | | | | | | | | |
|  | | | | | |  | | | | | | | | | | | | | | | |  | | | | | | | | | | | | | | | | | | | |
|  |  | | | | | | | | | | | | | | | | | | | | | | | | | | | | | | | | | | | | | | | | |
|  | |  | | |  | | | | | | | | | | | | | | | | |  |  | |  | | | |  |  |  |  |  |  |  |  |  |  |  |  |  |
|  | | |  | **Table S1: One-Way Sensitivity Analyses: Costs, cont.** | | | | | | | | | |  |  |  |  |  |  |  |  | | |  | |  |  | | | | | | | | | | | | | | |
|  | | |  |  | | |  |  |  |  |  |  |  |  |  |  |  |  |  |  |  | | |  | |  |  | | | | | | | | | | | | | | |
|  | | |  | **Alcohol Outcomes** | | | |  |  |  |  |  |  |  |  |  |  |  |  |  |  | | | | | | | | | | | | | | | | | | | | |
|  | | |  |  | | |  |  | **ICER (∆C/∆E, $), BL to 4mo** | | | | | | | | | | |  |  | | | | | | | | | | | | | | | | | | | | |
|  | | |  |  | | |  |  | Cost per Drink Avoided^h^ | |  | Cost per Reduced Drink per Day^h^ | |  | Cost per Heavy Drinker Prevented^i^ | |  | Cost per Abstainer^j^ | |  |  | | | | | | | | | | | | | | | | | | | | |
|  | | |  | Incremental Cost, B - A | | | Incremental Cost, C - B |  | WWE | 4ES |  | WWE | 4ES |  | WWE | 4ES |  | WWE | 4ES |  |  | | | | | | | | | | | | | | | | | | | | |
|  | | |  | ($144.45) | | | ($942.3) |  | (D) | ($233) |  | (D) | ($608) |  | (D) | ($31,410) |  | ($3,611) | (D) |  |  | | | | | | | | | | | | | | | | | | | | |
|  | | |  | $100 | | | $549^d^ |  | D | $136 |  | D | $354 |  | D | $18,300 |  | $2,500 | D |  |  | | | | | | | | | | | | | | | | | | | | |
|  | | |  | $200 | | | $759^e^ |  | D | $187 |  | D | $490 |  | D | $25,300 |  | $5,000 | D |  |  | | | | | | | | | | | | | | | | | | | | |
|  | | |  | $500 | | | $777^f^ |  | D | $192 |  | D | $501 |  | D | $25,900 |  | $12,500 | D |  |  | | | | | | | | | | | | | | | | | | | | |
|  | | |  | $1,000 | | | $1,233^g^ |  | D | $304 |  | D | $795 |  | D | $41,100 |  | $25,000 | D |  |  | | | | | | | | | | | | | | | | | | | | |
|  | | |  |  | | |  |  |  |  |  |  |  |  |  |  |  |  |  |  |  | | | | | | | | | | | | | | | | | | | | |
|  | | |  |  | | |  |  | **ICER (∆C/∆E, $), BL to 12mo** | | | | | | | | | | |  |  | | | | | | | | | | | | | | | | | | | | |
|  | | |  |  | | |  |  | Cost per Drink Avoided^h^ | |  | Cost per Reduced Drink per Day^h^ | |  | Cost per Heavy Drinker Prevented^i^ | |  | Cost per Abstainer^j^ | |  |  | | | | | | | | | | | | | | | | | | | | |
|  | | |  | Incremental Cost, B - A | | | Incremental Cost, C - B |  | WWE | 4ES |  | WWE | 4ES |  | WWE | 4ES |  | WWE | 4ES |  |  | | | | | | | | | | | | | | | | | | | | |
|  | | |  | ($144.45) | | | ($942.3) |  | ($28) | ($688) |  | ($147) | ($1,273) |  | ($4,815) | ($94,230) |  | ($7,223) | (D) |  |  | | | | | | | | | | | | | | | | | | | | |
|  | | |  | $100 | | | $549^d^ |  | $19 | $401 |  | $102 | $742 |  | $3,333 | $54,900 |  | $5,000 | D |  |  | | | | | | | | | | | | | | | | | | | | |
|  | | |  | $200 | | | $759^e^ |  | $39 | $554 |  | $204 | $1,026 |  | $6,667 | $75,900 |  | $10,000 | D |  |  | | | | | | | | | | | | | | | | | | | | |
|  | | |  | $500 | | | $777^f^ |  | $97 | $567 |  | $510 | $1,050 |  | $16,667 | $77,700 |  | $25,000 | D |  |  | | | | | | | | | | | | | | | | | | | | |
|  | | |  | $1,000 | | | $1,233^g^ |  | $194 | $900 |  | $1,020 | $1,666 |  | $33,333 | $123,300 |  | $50,000 | D |  |  | | | | | | | | | | | | | | | | | | | | |
|  | | |  |  | | |  |  |  |  |  |  |  |  |  |  |  |  |  |  |  | | | | | | | | | | | | | | | | | | | | |
|  | | |  | Abbreviations: WWE, NIDA’s Standard Intervention plus Well Woman Exam; 4ES, WWE plus four educational sessions; ICER, incremental cost effectiveness ratio, which is the  difference in cost divided by the difference in effectiveness as compared with the next least costly intervention, and indicates cost per additional outcome achieved; D, dominated,  which indicates that the intervention is more costly and less effective than the alternative; ED, extended dominated, which indicates that the next alternative is more costly, more  effective and has a better ICER. Base case values given in parentheses. | | | | | | | | | | | | | | | | | | | | | |  | |  | | | | | | | | | | | | | |
|  | | |  | ^*^  ^a^ Sensitivity analyses figures are taken from Ruger et al. (2010). Base case values given in parentheses. | | | | | | | | | | | | |  |  |  |  |  | | | | |  | |  | | | | | | | | | | | | | |
|  | | |  | ^b^ Past 30 days. | | |  |  |  |  |  |  |  |  |  |  |  |  |  |  |  | | | | |  | |  | | | | | | | | | | | | | |
|  | | |  | ^c^ Per day, past 30 days. | | | |  |  |  |  |  |  |  |  |  |  |  |  |  |  | | | | |  | |  | | | | | | | | | | | | | |
|  | | |  | ^d^ 50% decrease in building rental/utilities. | | | | | |  | | | |  |  |  |  |  |  |  |  | | | | |  | |  | | | | | | | | | | | | | |
|  | | |  | ^e^ If each session has five people. | | | | | |  |  |  |  |  |  |  |  |  |  |  |  | | | | |  | |  | | | | | | | | | | | | | |
|  | | |  | ^f^ 25% decrease in building rental/utilities. | | | | | |  |  |  |  |  |  |  |  |  |  |  |  | | | | |  | |  | | | | | | | | | | | | | |
|  | | |  | ^g^ If each session has three people. | | | | | |  |  |  |  |  |  |  |  |  |  |  |  | | | | |  | |  | | | | | | | | | | | | | |
|  | | |  | ^h^ Past 7 days. | | |  |  |  |  |  |  |  |  |  |  |  |  |  |  |  | | | | |  | |  | | | | | | | | | | | | | |
|  | | |  | ^i^ Past 7 days. "Heavy drinker" includes patients who drink > 4 drinks per day of the days participants drank alcohol. | | | | | | | | | | | | | |  |  |  |  | | | | |  | |  | | | | | | | | | | | | | |
|  | | |  | ^j^ Past 30 days. | | |  |  |  |  |  |  |  |  |  |  |  |  |  |  |  | | | | |  | |  | | | | | | | | | | | | | |
