# Supplementary material for: Cost-Effectiveness of Peer-Delivered Interventions for Cocaine and Alcohol Abuse among Women: A Randomized Controlled Trial
Source: PLoS One. 2012 Mar 20;7(3):e33594. doi: 10.1371/journal.pone.0033594 (PMC3308978; doi:10.1371/journal.pone.0033594)
Supplement: Table S2 — One-way Sensitivity Analyses: Outcomes. (DOC) [file pone.0033594.s005.doc]

**Table S2: One-Way Sensitivity Analyses: Outcomes**

|  |  |  |  |  |  |  |  |
| --- | --- | --- | --- | --- | --- | --- | --- |
|  |  | | | | | |  |
|  |  |  |  |  |  |  |  |
|  |  | **ICER (∆C/∆E, $), 4mo** | |  | **ICER (∆C/∆E, $), 12mo** | |  |
|  | **Parameter Varied^a^** | **B-A** | **C-B** |  | **B-A** | **C-B** |  |
|  |  | (BC) | (BC) |  | (BC) | (BC) |  |
|  | **Cocaine Use** |  |  |  |  |  |  |
|  | **Occasions^b^ Used, past 30 days** | |  |  |  |  |  |
|  | **Varying B^c^** | (D) | ($146) |  | ($336) | ($43) |  |
|  | 12 occasions (+51%, +65%) | $11 | D |  | $6 | D |  |
|  | 15 occasions (+39%, +57%) | $15 | D |  | $7 | $419 |  |
|  | +5% | **$123** | $181 |  | $67 | $47 |  |
|  | -5% | *D* | $123 |  | **D** | $40 |  |
|  | **Varying C^c^** | (D) | ($146) |  | ($336) | ($43) |  |
|  | 12 occasions (+51%, +65%) | *D* | $50 |  | *$336* | $33 |  |
|  | 15 occasions (+39%, +29%) | *D* | $59 |  | *$336* | $36 |  |
|  | -5% | *D* | $181 |  | *$336* | $45 |  |
|  | -30% | *D* | **D** |  | *$336* | $58 |  |
|  | -115% | *D* | D |  | *$336* | **D** |  |
|  | **Episodes per Day, past 30 days** | |  |  |  |  |  |
|  | **Varying B^c^** | ($498) | ($3,490) |  | (D) | ($1,059) |  |
|  | 1 episode (+49%, +61%) | $116 | D |  | $97 | D |  |
|  | 0.5 episode (+74%, +80%) | $83 | D |  | $73 | D |  |
|  | +5% | $373 | $5,463 |  | **$2,491** | $1,237 |  |
|  | -5% | $750 | $2,564 |  | *D* | $926 |  |
|  | -15% | **D** | $1,675 |  | *D* | $740 |  |
|  | **Varying C^c^** | ($498) | ($3,490) |  | (D) | ($1,059) |  |
|  | 1 episode (+48%, +48%) | *$498* | $792 |  | *D* | $524 |  |
|  | 0.5 episode (+74%, +74%) | *$498* | $558 |  | *D* | $410 |  |
|  | -5% | *$498* | $5,416 |  | *D* | $1,186 |  |
|  | -15% | *$498* | **D** |  | *D* | $1,561 |  |
|  | -50% | *$498* | D |  | *D* | **D** |  |
|  | **Cocaine Free Days, past 30 days** | |  |  |  |  |  |
|  | **Varying B^c^** | (D) | (D) |  | (D) | ($504) |  |
|  | 3wk abstinence (+12%, +26%) | $51 | D |  | $26 | D |  |
|  | 85% days free (+2%, +15%) | $431 | D |  | $47 | D |  |
|  | +5% | **$135** | D |  | **$197** | $1,229 |  |
|  | **Varying C^c^** | (D) | (D) |  | (D) | ($504) |  |
|  | 3wk abstinence (+14%, +15%) | *D* | $349 |  | *D* | $168 |  |
|  | 85% days free (+4%, +5%) | *D* | $4,712 |  | *D* | $303 |  |
|  | +5% | *D* | **$1,678** |  | *D* | $306 |  |
|  | -5% | *D* | D |  | *D* | $1,434 |  |
|  | -10% | *D* | D |  | *D* | **D** |  |
|  | **Abstaining,^d^ past 30 days** |  |  |  |  |  |  |
|  | **Varying B^e^** | ($14,445) | ($18,846) |  | (D) | (D) |  |
|  | +5% | $4,514 | $33,654 |  | **$8,755** | D |  |
|  | -5% | **D** | $13,088 |  | *D* | D |  |

|  | **Table S2: One-Way Sensitivity Analyses: Outcomes, cont.** | | | | | |  |
| --- | --- | --- | --- | --- | --- | --- | --- |
|  |  | **ICER (∆C/∆E, $), 4mo** | |  | **ICER (∆C/∆E, $), 12mo** | |  |
|  | **Parameter Varied^a^** | **B-A** | **C-B** |  | **B-A** | **C-B** |  |
|  |  | (BC) | (BC) |  | (BC) | (BC) |  |

|  | **Varying C^e^** | ($14,445) | ($18,846) |  | (D) | (D) |  |
| --- | --- | --- | --- | --- | --- | --- | --- |
|  | +5% | *$14,445* | $12,564 |  | *D* | D |  |
|  | +10% | *$14,445* | $9,423 |  | *D* | **$78,525** |  |
|  | -5% | *$14,445* | $37,692 |  | *D* | D |  |
|  | -10% | *$14,445* | **D** |  | *D* | D |  |
|  | **Abstaining,^d^ past 4 months** |  |  |  |  |  |  |
|  | **Varying B^e^** | ($7,223) | ($18,846) |  | ($3,611) | (D) |  |
|  | -5% | $48,150 | $14,064 |  | $5,896 | D |  |
|  | -10% | **D** | $11,218 |  | $16,050 | $942,300 |  |
|  | -15% | D | $9,330 |  | **D** | $57,109 |  |
|  | **Varying C^e^** | ($7,223) | ($18,846) |  | ($3,611) | (D) |  |
|  | +5% | *$7,223* | $13,366 |  | *$3,611* | D |  |
|  | +15% | *$7,223* | $8,451 |  | *$3,611* | **$62,820** |  |
|  | -5% | *$7,223* | $31,942 |  | *$3,611* | D |  |
|  | -15% | *$7,223* | **D** |  | *$3,611* | D |  |
|  | **Alcohol Consumption** |  |  |  |  |  |  |
|  | **Drinks, past 7 days** |  |  |  |  |  |  |
|  | **Varying B^e^** | (D) | ($233) |  | ($28) | ($688) |  |
|  | +5% | *D* | $288 |  | $25 | $1,407 |  |
|  | +10% | **$347** | $379 |  | $22 | **D** |  |
|  | -10% | *D* | $168 |  | $39 | $340 |  |
|  | -40% | *D* | $91 |  | **D** | $135 |  |
|  | **Varying C^e^** | (D) | ($233) |  | ($28) | ($688) |  |
|  | -10% | *D* | $331 |  | *$28* | $14,064 |  |
|  | -15% | *D* | $419 |  | *$28* | **D** |  |
|  | -35% | *D* | **D** |  | *$28* | D |  |
|  | **Drinks per Day, past 7 days** |  |  |  |  |  |  |
|  | **Varying B^c^** | (D) | ($608) |  | ($147) | ($1,273) |  |
|  | 4 drinks (+10%, -4%) | *D* | $834 |  | $176 | $1,047 |  |
|  | 1 drink (+77%, +74%) | $57 | D |  | $38 | D |  |
|  | +5% | *D* | $709 |  | $123 | $1,720 |  |
|  | +20% | *D* | $1,415 |  | $83 | **D** |  |
|  | +25% | **$672** | $2,118 |  | $74 | D |  |
|  | -30% | *D* | $328 |  | **D** | $498 |  |
|  | **Varying C^c^** | (D) | ($608) |  | ($147) | ($1,273) |  |
|  | 4 drinks (+26%, +17%) | *D* | $1,291 |  | *$147* | $6,282 |  |
|  | 1 drink (+69%, +71%) | *D* | $253 |  | *$147* | $299 |  |
|  | -5% | *D* | $677 |  | *$147* | $1,655 |  |
|  | -25% | *D* | $1,248 |  | *$147* | **D** |  |
|  | -50% | *D* | **D** |  | *$147* | D |  |
|  | **Preventing Heavy Drinkers, past 7 days** | | |  |  |  |  |
|  | **Varying B^e^** | (D) | ($31,410) |  | ($4,815) | ($94,230) |  |
|  | +5% | **$48,150** | D |  | $2,349 | D |  |
|  | -5% | D | $14,957 |  | **D** | $22,706 |  |

|  | **Table S2: One-Way Sensitivity Analyses: Outcomes, cont.** | | | | | | | | | | |  | |
| --- | --- | --- | --- | --- | --- | --- | --- | --- | --- | --- | --- | --- | --- |
|  |  | | **ICER (∆C/∆E, $), 4mo** | | | |  | | **ICER (∆C/∆E, $), 12mo** | | | |  |
|  | **Parameter Varied^a^** | | **B-A** | | **C-B** | |  | | **B-A** | | **C-B** | |  |
|  |  | | (BC) | | (BC) | |  | | (BC) | | (BC) | |  |
|  | **Varying C^e^** | (D) | | ($31,410) | |  | | ($4,815) | | ($94,230) | |  | |
|  | +5% | D | | $14,839 | |  | | $4,815 | | $22,983 | |  | |
|  | -5% | D | | **D** | |  | | $4,815 | | **D** | |  | |
|  | **Abstaining,^f^ past 30 days** |  | |  | |  | |  | |  | |  | |
|  | **Varying B^e^** | ($3,611) | | (D) | |  | | ($7,223) | | (D) | |  | |
|  | -5% | $7,408 | | D | |  | | $57,780 | | D | |  | |
|  | -10% | **D** | | D | |  | | **D** | | $62,820 | |  | |
|  | **Varying C^e^** | ($3,611) | | (D) | |  | | ($7,223) | | (D) | |  | |
|  | +5% | *$3,611* | | D | |  | | *$7,223* | | D | |  | |
|  | +15% | *$3,611* | | **$628,200** | |  | | *$7,223* | | **$24,475** | |  | |
|  |  |  | |  | |  | |  | |  | |  | |
|  | Abbreviations: ICER, incremental cost effectiveness ratio, which is the difference in cost divided by the difference in effectiveness as compared with the next least costly intervention, and indicates cost per additional outcome achieved; D, dominated, which indicates that the intervention is more costly and less effective than the alternative; BC, base case; A, SI intervention; B, SI+WWE intervention; C, SI+WWE+4ES intervention. | | | | | | | | | | |  | |
|  | Bold indicates the point at which the intervention switches from being dominated to not being dominated, or vice versa. Italics indicate equivalence to the base case value because changing the cost of C does not impact the B-A ICER. | | | | | | | | | | |  | |
|  | ^a^ Percentage varied indicates percentage change of base case value for indicated parameter only, whereas base case of other two respective arms remain the same. A negative percentage change indicates less effective intervention than the base case, which may or may not indicate a mean lower than the base case (see Table 2). A positive percentage change indicates a more effective intervention than base case. Included in parentheses next to the clinically significant changes are the corresponding percentage changes. The first percentage in the parentheses is the change relative to the 4 month value and the second percentage is the change relative to the 12 month value. | | | | | | | | | | |  | |
|  | ^b^ Occasions = days used * times per day. |  | |  | |  | |  | |  | |  | |
|  | ^c^ Varying base case values through clinically significant ranges and statistically significant switching points to assess changes in CEA threshold. | | | | | | | | | | |  | |
|  | ^d^ Proportion of patients abstaining from cocaine. | | | | | | | | | | |  | |
|  | ^e^ Varying base case values through ranges of statistically significant switching points to assess changes in CEA threshold. | | | | | | | | | | |  | |
|  | ^f^ Proportion of patients abstaining from alcohol. | | | | |  | |  | |  | |  | |
