# Supplementary material for: Cost-Effectiveness of Peer-Delivered Interventions for Cocaine and Alcohol Abuse among Women: A Randomized Controlled Trial
Source: PLoS One. 2012 Mar 20;7(3):e33594. doi: 10.1371/journal.pone.0033594 (PMC3308978; doi:10.1371/journal.pone.0033594)
Supplement: Table S3 — Two-way Sensitivity Analyses. (DOC) [file pone.0033594.s006.doc]

**Table S3: Two-Way Sensitivity Analyses**

|  |  | |  | | |  |  |  |  |  |  |  |
| --- | --- | --- | --- | --- | --- | --- | --- | --- | --- | --- | --- | --- |
|  | | | | | | |  |  |  |  |  |  |
|  |  | |  | | |  |  |  |  |  |  |  |
|  | **ICER (∆C/∆E, $), 4mo** | | | | | | | | | | | |
|  | **B - A** | | | | | | |  | **C - B** | | | |
| **Incremental Cost** | $100 | | $200 | | | $500 | $1,000 |  | $549^a^ | $759^b^ | $777^c^ | $1,233^d^ |
| **Parameter Varied^e^** |  | |  | | |  |  |  |  |  |  |  |
| **Cocaine Use** |  | |  | |  | |  |  |  |  |  |  |
| **Occasions^f^ Used, past 30 days** |  | | | | | |  |  |  |  |  |  |
| **Varying B^g^** |  | |  | |  | |  |  |  |  |  |  |
| 12 occasions (+51%) | $8 | | $16 | | | $40 | $79 |  | D | D | D | D |
| 15 occasions (+39%) | $10 | | $21 | | | $52 | $104 |  | D | D | D | D |
| +5% | $85 | | $170 | | | $426 | $851 |  | $105 | $146 | $149 | $237 |
| -5% | D | | D | | | D | D |  | $72 | $99 | $101 | $161 |
| **Varying C^g^** |  | |  | | |  |  |  |  |  |  |  |
| 12 occasions (+51%) | *D* | | *D* | | | *D* | *D* |  | $29 | $40 | $41 | $65 |
| 15 occasions (+39%) | *D* | | *D* | | | *D* | *D* |  | $34 | $47 | $48 | $77 |
| -5% | *D* | | *D* | | | *D* | *D* |  | $105 | $146 | $149 | $237 |
| -30% | *D* | | *D* | | | *D* | *D* |  | D | D | D | D |
| -115% | *D* | | *D* | | | *D* | *D* |  | D | D | D | D |
| **Episodes per Day, past 30 days** |  | | | | | |  |  |  |  |  |  |
| **Varying B^g^** |  | |  |  | | |  |  |  |  |  |  |
| 1 episode (+49%) | $81 | | $161 | | | $403 | $806 |  | D | D | D | D |
| 0.5 episode (+74%) | $57 | | $115 | | | $287 | $575 |  | D | D | D | D |
| +5% | $258 | | $516 | | | $1,290 | $2,581 |  | $3,183 | $4,400 | $4,504 | $7,148 |
| -5% | $519 | | $1,039 | | | $2,597 | $5,195 |  | $1,494 | $2,065 | $2,114 | $3,355 |
| -15% | D | | D | | | D | D |  | $976 | $1,349 | $1,381 | $2,192 |
| **Varying C^g^** |  | |  | | |  |  |  |  |  |  |  |
| 1 episode (+48%) | *$345* | | *$690* | | | *$1,724* | *$3,448* |  | $461 | $638 | $653 | $1,036 |
| 0.5 episode (+74%) | *$345* | | *$690* | | | *$1,724* | *$3,448* |  | $325 | $449 | $460 | $730 |
| -5% | *$345* | | *$690* | | | *$1,724* | *$3,448* |  | $3,155 | $4,362 | $4,466 | $7,086 |
| -15% | *$345* | | *$690* | | | *$1,724* | *$3,448* |  | D | D | D | D |
| -50% | *$345* | | *$690* | | | *$1,724* | *$3,448* |  | D | D | D | D |
| **Cocaine Free Days, past 30 days** |  | | | | |  |  |  |  |  |  |  |
| **Varying B^g^** |  |  | | | |  |  |  |  |  |  |  |
| 3wk abstinence (+12%) | $35 | | $70 | | | $175 | $351 |  | D | D | D | D |
| 85% days free (+2%) | $286 | | $571 | | | $1,429 | $2,857 |  | D | D | D | D |
| +5% | $94 | | $187 | | | $468 | $936 |  | D | D | D | D |

| **Table S3: Two-Way Sensitivity Analyses, cont.** | | | | | |  |  |  |  |  |
| --- | --- | --- | --- | --- | --- | --- | --- | --- | --- | --- |
|  | **ICER (∆C/∆E, $), 4mo** | | | | | | | | | |
|  | **B – A** | | | | |  | **C - B** | | | |
| **Incremental Cost** | $100 | $200 | | $500 | $1,000 |  | $549^a^ | $759^b^ | $777^c^ | $1,233^d^ |
| **Parameter Varied^e^** |  |  | |  |  |  |  |  |  |  |
| **Varying C^g^** |  |  | |  |  |  |  |  |  |  |
| 3wk abstinence (+14%) | *D* | *D* | | *D* | *D* |  | $203 | $281 | $288 | $457 |
| 85% days free (+4%) | *D* | *D* | | *D* | *D* |  | $2,745 | $3,795 | $3,885 | $6,165 |
| +5% | *D* | *D* | | *D* | *D* |  | $978 | $1,352 | $1,384 | $2,196 |
| -5% | *D* | *D* | | *D* | *D* |  | D | D | D | D |
| -10% | *D* | *D* | | *D* | *D* |  | D | D | D | D |
| **Abstaining,^h^ past 30 days** |  |  | |  |  |  |  |  |  |  |
| **Varying B^i^** |  |  | |  |  |  |  |  |  |  |
| +5% | $3,125 | $6,250 | | $15,625 | $31,250 |  | $19,607 | $27,107 | $27,750 | $44,036 |
| -5% | D | D | | D | D |  | $7,625 | $10,542 | $10,792 | $17,125 |
| **Varying C^i^** |  |  | |  |  |  |  |  |  |  |
| +5% | *$10,000* | *$20,000* | | *$50,000* | *$100,000* |  | $7,320 | $10,120 | $10,360 | $16,440 |
| +10% | *$10,000* | *$20,000* | | *$50,000* | *$100,000* |  | $5,490 | $7,590 | $7,770 | $12,330 |
| -5% | *$10,000* | *$20,000* | | *$50,000* | *$100,000* |  | $21,960 | $30,360 | $31,080 | $49,320 |
| -10% | *$10,000* | *$20,000* | | *$50,000* | *$100,000* |  | D | D | D | D |
| **Abstaining,^h^ past 4 months** | |  |  | |  |  |  |  |  |  |
| **Varying B^i^** |  |  | |  |  |  |  |  |  |  |
| -5% | $33,333 | $66,667 | | $166,667 | $333,333 |  | $8,194 | $11,328 | $11,597 | $18,403 |
| -15% | D | D | | D | D |  | $5,436 | $7,515 | $7,693 | $12,208 |
| **Varying C^i^** |  |  | |  |  |  |  |  |  |  |
| +5% | *$5,000* | *$10,000* | | *$25,000* | *$50,000* |  | $7,787 | $10,766 | $11,021 | $17,489 |
| +15% | *$5,000* | *$10,000* | | *$25,000* | *$50,000* |  | $4,924 | $6,807 | $6,969 | $11,058 |
| -5% | *$5,000* | *$10,000* | | *$25,000* | *$50,000* |  | $18,610 | $25,729 | $26,339 | $41,797 |
| -15% | *$5,000* | *$10,000* | | *$25,000* | *$50,000* |  | D | D | D | D |
| **Alcohol Consumption** |  |  | |  |  |  |  |  |  |  |
| **Drinks, past 7 days** |  |  | |  |  |  |  |  |  |  |
| **Varying B^i^** |  |  | |  |  |  |  |  |  |  |
| +5% | D | D | | D | D |  | $168 | $232 | $238 | $377 |
| +10% | $240 | $481 | | $1,202 | $2,404 |  | $221 | $306 | $313 | $496 |
| -10% | D | D | | D | D |  | $98 | $135 | $138 | $220 |
| -40% | D | D | | D | D |  | $53 | $74 | $75 | $120 |
| **Varying C^i^** |  |  | |  |  |  |  |  |  |  |
| -10% | *D* | *D* | | *D* | *D* |  | $193 | $266 | $273 | $433 |
| -15% | *D* | *D* | | *D* | *D* |  | $244 | $337 | $345 | $548 |
| -35% | *D* | *D* | | *D* | *D* |  | D | D | D | D |

| **Table S3: Two-Way Sensitivity Analyses, cont.** | | | |  |  |  |  |  |  |
| --- | --- | --- | --- | --- | --- | --- | --- | --- | --- |
|  | **ICER (∆C/∆E, $), 4mo** | | | | | | | | |
|  | **B - A** | | | |  | **C - B** | | | |
| **Incremental Cost** | $100 | $200 | $500 | $1,000 |  | $549^a^ | $759^b^ | $777^c^ | $1,233^d^ |
| **Parameter Varied^e^** |  | |  |  |  |  |  |  |  |
| **Drinks per Day, past 7 days** |  | |  |  |  |  |  |  |  |
| **Varying B^g^** |  |  |  |  |  |  |  |  |  |
| 4 drinks (+10%) | D | D | D | D |  | $486 | $672 | $688 | $1,091 |
| 1 drink (+77%) | $40 | $79 | $198 | $395 |  | D | D | D | D |
| +5% | D | D | D | D |  | $413 | $571 | $585 | $928 |
| +20% | D | D | D | D |  | $824 | $1,140 | $1,167 | $1,851 |
| +25% | $465 | $930 | $2,326 | $4,651 |  | $1,234 | $1,706 | $1,746 | $2,771 |
| -30% | D | D | D | D |  | $191 | $264 | $270 | $429 |
| **Varying C^g^** |  |  |  |  |  |  |  |  |  |
| 4 drinks (+26%) | *D* | *D* | *D* | *D* |  | $752 | $1,040 | $1,064 | $1,689 |
| 1 drink (+69%) | *D* | *D* | *D* | *D* |  | $147 | $203 | $208 | $331 |
| -5% | *D* | *D* | *D* | *D* |  | $395 | $546 | $559 | $886 |
| -25% | *D* | *D* | *D* | *D* |  | $727 | $1,005 | $1,029 | $1,633 |
| -50% | *D* | *D* | *D* | *D* |  | D | D | D | D |
| **Preventing Heavy Drinkers, past 7 days** |  |  |  |  |  |  |  |  |  |
| **Varying B^i^** |  |  |  |  |  |  |  |  |  |
| +5% | $33,333 | $66,667 | $166,667 | $333,333 |  | D | D | D | D |
| -5% | D | D | D | D |  | $8,714 | $12,048 | $12,333 | $19,571 |
| **Varying C^i^** |  |  |  |  |  |  |  |  |  |
| +5% | *D* | *D* | *D* | *D* |  | $8,646 | $11,953 | $12,236 | $19,417 |
| -5% | *D* | *D* | *D* | *D* |  | D | D | D | D |
| **Abstaining,^j^ past 30 days** |  |  |  |  |  |  |  |  |  |
| **Varying B^i^** |  |  |  |  |  |  |  |  |  |
| -5% | $5,128 | $10,256 | $25,641 | $51,282 |  | D | D | D | D |
| -10% | D | D | D | D |  | D | D | D | D |
| **Varying C^i^** |  |  |  |  |  |  |  |  |  |
| +5% | *$2,500* | *$5,000* | *$12,500* | *$25,000* |  | D | D | D | D |
| +15% | *$2,500* | *$5,000* | *$12,500* | *$25,000* |  | $366,000 | $506,000 | $518,000 | $822,000 |

**Table S3: Two-Way Sensitivity Analyses, cont.**

|  | | | | | | | | | |
| --- | --- | --- | --- | --- | --- | --- | --- | --- | --- |
|  | **ICER (∆C/∆E, $), 12mo** | | | | | | | | |
| **Incremental Cost** | **B - A** | | | |  | **C - B** | | | |
|  | $100 | $200 | $500 | $1,000 |  | $549^a^ | $759^b^ | $777^c^ | $1,233^d^ |
| **Parameter Varied^e^** |  |  |  |  |  |  |  |  |  |
| **Cocaine Use** |  |  |  |  |  |  |  |  |  |
| **Occasions^f^ Used, past 30 days** | | |  |  |  |  |  |  |  |
| **Varying B^g^** |  |  |  |  |  |  |  |  |  |
| 12 occasions (+65%) | $4 | $9 | $22 | $43 |  | D | D | D | D |
| 15 occasions (+57%) | $5 | $10 | $25 | $50 |  | $244 | $337 | $345 | $548 |
| +5% | $46 | $93 | $231 | $463 |  | $27 | $38 | $39 | $61 |
| -5% | D | D | D | D |  | $23 | $32 | $33 | $52 |
| **Varying C^g^** |  |  |  |  |  |  |  |  |  |
| 12 occasions (+65%) | *$233* | *$465* | *$1,163* | *$2,326* |  | $19 | $26 | $27 | $43 |
| 15 occasions (+29%) | *$233* | *$465* | *$1,163* | *$2,326* |  | $21 | $29 | $30 | $48 |
| -5% | *$233* | *$465* | *$1,163* | *$2,326* |  | $26 | $36 | $37 | $59 |
| -30% | *$233* | *$465* | *$1,163* | *$2,326* |  | $34 | $47 | $48 | $76 |
| -115% | *$233* | *$465* | *$1,163* | *$2,326* |  | D | D | D | D |
| **Episodes per Day, past 30 days** |  |  |  |  |  |  |  |  |  |
| **Varying B^g^** |  |  |  |  |  |  |  |  |  |
| 1 episode (+61%) | $67 | $134 | $336 | $671 |  | D | D | D | D |
| 0.5 episode (+80%) | $50 | $101 | $251 | $503 |  | D | D | D | D |
| +5% | $1,724 | $3,448 | $8,621 | $17,241 |  | $720 | $996 | $1,020 | $1,618 |
| -5% | D | D | D | D |  | $539 | $746 | $763 | $1,211 |
| -15% | D | D | D | D |  | $431 | $596 | $610 | $968 |
| **Varying C^g^** |  |  |  |  |  |  |  |  |  |
| 1 episode (+48%) | *D* | *D* | *D* | *D* |  | $305 | $422 | $432 | $685 |
| 0.5 episode (+74%) | *D* | *D* | *D* | *D* |  | $239 | $330 | $338 | $536 |
| -5% | *D* | *D* | *D* | *D* |  | $691 | $955 | $978 | $1,552 |
| -15% | *D* | *D* | *D* | *D* |  | $910 | $1,258 | $1,287 | $2,043 |
| -50% | *D* | *D* | *D* | *D* |  | D | D | D | D |
| **Cocaine Free Days, past 30 days** |  |  |  |  |  |  |  |  |  |
| **Varying B^g^** |  |  |  |  |  |  |  |  |  |
| 3wk abstinence (+26%) | $18 | $36 | $90 | $180 |  | D | D | D | D |
| 85% days free (+15%) | $33 | $65 | $163 | $326 |  | D | D | D | D |
| +5% | $136 | $273 | $682 | $1,364 |  | $716 | $990 | $1,013 | $1,608 |
| **Varying C^g^** |  |  |  |  |  |  |  |  |  |
| 3wk abstinence (+15%) | *D* | *D* | *D* | *D* |  | $98 | $135 | $139 | $220 |
| 85% days free (+5%) | *D* | *D* | *D* | *D* |  | $177 | $244 | $250 | $396 |
| +5% | *D* | *D* | *D* | *D* |  | $178 | $246 | $252 | $400 |
|  |  |  |  |  |  |  |  |  |  |
| **Table S3: Two-Way Sensitivity Analyses, cont.** | | | |  |  |  |  |  |  |
|  | **ICER (∆C/∆E, $), 12mo** | | | | | | | | |
| **Incremental Cost** | **B - A** | | | |  | **C - B** | | | |
|  | $100 | $200 | $500 | $1,000 |  | $549^a^ | $759^b^ | $777^c^ | $1,233^d^ |
| **Parameter Varied^e^** |  |  |  |  |  |  |  |  |  |
| -5% | *D* | *D* | *D* | *D* |  | $836 | $1,155 | $1,183 | $1,877 |
| -10% | *D* | *D* | *D* | *D* |  | D | D | D | D |
| **Abstaining,^h^ past 30 days** |  |  |  |  |  |  |  |  |  |
| **Varying B^i^** |  |  |  |  |  |  |  |  |  |
| +5% | $6,061 | $12,121 | $30,303 | $60,606 |  | D | D | D | D |
| -5% | D | D | D | D |  | D | D | D | D |
| **Varying C^i^** |  |  |  |  |  |  |  |  |  |
| +5% | *D* | *D* | *D* | *D* |  | D | D | D | D |
| +10% | *D* | *D* | *D* | *D* |  | $45,750 | $63,250 | $64,750 | $102,750 |
| -5% | *D* | *D* | *D* | *D* |  | D | D | D | D |
| -10% | *D* | *D* | *D* | *D* |  | D | D | D | D |
| **Abstaining,^h^ past 4 months** |  |  |  |  |  |  |  |  |  |
| **Varying B^i^** |  |  |  |  |  |  |  |  |  |
| -5% | $4,082 | $8,163 | $20,408 | $40,816 |  | D | D | D | D |
| -15% | D | D | D | D |  | D | D | D | D |
| **Varying C^i^** |  |  |  |  |  |  |  |  |  |
| 5% | *$2,500* | *$5,000* | *$12,500* | *$25,000* |  | D | D | D | D |
| 15% | *$2,500* | *$5,000* | *$12,500* | *$25,000* |  | $36,600 | $50,600 | $51,800 | $82,200 |
| -5% | *$2,500* | *$5,000* | *$12,500* | *$25,000* |  | D | D | D | D |
| -15% | *$2,500* | *$5,000* | *$12,500* | *$25,000* |  | D | D | D | D |
| **Alcohol Consumption** |  |  |  |  |  |  |  |  |  |
| **Drinks, past 7 days** |  |  |  |  |  |  |  |  |  |
| **Varying B^i^** |  |  |  |  |  |  |  |  |  |
| +5% | $17 | $34 | $85 | $171 |  | $820 | $1,134 | $1,161 | $1,842 |
| +10% | $15 | $31 | $76 | $153 |  | D | D | D | D |
| -10% | $27 | $53 | $133 | $267 |  | $198 | $274 | $280 | $445 |
| -40% | D | D | D | D |  | $79 | $109 | $111 | $177 |
| **Varying C^i^** |  |  |  |  |  |  |  |  |  |
| -10% | *$19* | *$39* | *$97* | *$194* |  | $8,194 | $11,328 | $11,597 | $18,403 |
| -15% | *$19* | *$39* | *$97* | *$194* |  | D | D | D | D |
| -35% | *$19* | *$39* | *$97* | *$194* |  | D | D | D | D |
| **Drinks per Day, past 7 days** |  |  |  |  |  |  |  |  |  |
| **Varying B^g^** |  |  |  |  |  |  |  |  |  |
| 4 drinks (-4%) | $122 | $244 | $610 | $1,220 |  | $610 | $843 | $863 | $1,370 |
| 1 drink (+74%) | $26 | $52 | $131 | $262 |  | D | D | D | D |
| +5% | $85 | $171 | $427 | $853 |  | $1,002 | $1,385 | $1,418 | $2,250 |
|  |  |  |  |  |  |  |  |  |  |
| **Table S3: Two-Way Sensitivity Analyses, cont.** | | |  |  |  |  |  |  |  |
|  | **ICER (∆C/∆E, $), 12mo** | | | | | | | | |
| **Incremental Cost** | **B - A** | | | |  | **C - B** | | | |
|  | $100 | $200 | $500 | $1,000 |  | $549^a^ | $759^b^ | $777^c^ | $1,233^d^ |
| **Parameter Varied^e^** |  |  |  |  |  |  |  |  |  |
| +20% | $57 | $114 | $286 | $572 |  | D | D | D | D |
| +25% | $52 | $103 | $258 | $515 |  | D | D | D | D |
| -30% | D | D | D | D |  | $290 | $401 | $411 | $652 |
| **Varying C^g^** |  |  |  |  |  |  |  |  |  |
| 4 drinks (+17%) | *$102* | *$204* | *$510* | *$1,020* |  | $3,660 | $5,060 | $5,180 | $8,220 |
| 1 drink (+71%) | *$102* | *$204* | *$510* | *$1,020* |  | $174 | $241 | $247 | $391 |
| -5% | *$102* | *$204* | *$510* | *$1,020* |  | $964 | $1,333 | $1,364 | $2,165 |
| -25% | *$102* | *$204* | *$510* | *$1,020* |  | D | D | D | D |
| -50% | *$102* | *$204* | *$510* | *$1,020* |  | D | D | D | D |
| **Preventing Heavy Drinkers, past 7 days** |  |  |  |  |  |  |  |  |  |
| **Varying B^i^** |  |  |  |  |  |  |  |  |  |
| +5% | $1,626 | $3,252 | $8,130 | $16,260 |  | D | D | D | D |
| -5% | D | D | D | D |  | $13,229 | $18,289 | $18,723 | $29,711 |
| **Varying C^i^** |  |  |  |  |  |  |  |  |  |
| +5% | *$3,333* | *$6,667* | *$16,667* | *$33,333* |  | $13,390 | $18,512 | $18,951 | $30,073 |
| -5% | *$3,333* | *$6,667* | *$16,667* | *$33,333* |  | D | D | D | D |
| **Abstaining,^j^ past 30 days** |  |  |  |  |  |  |  |  |  |
| **Varying B^i^** |  |  |  |  |  |  |  |  |  |
| -5% | $40,000 | $80,000 | $200,000 | $400,000 |  | D | D | D | D |
| -10% | D | D | D | D |  | $36,600 | $50,600 | $51,800 | $82,200 |
| **Varying C^i^** |  |  |  |  |  |  |  |  |  |
| +5% | *$5,000* | *$10,000* | *$25,000* | *$50,000* |  | D | D | D | D |
| +15% | *$5,000* | *$10,000* | *$25,000* | *$50,000* |  | $14,260 | $19,714 | $20,182 | $32,026 |
| Abbreviations: ICER, incremental cost effectiveness ratio, which is the difference in cost divided by the difference in effectiveness as compared with the next least costly intervention and indicates cost per additional outcome achieved; D, dominated, which indicates that the intervention is more costly and less effective than the alternative; A, SI intervention; B, SI+WWE intervention; C, SI+WWE+4ES intervention. | | | | | | | | | |
| Italics indicate equivalence to the base case value because changing the cost of C does not impact the B-A ICER. | | | | | | | | | |
| ^a^ 50% decrease in building rental/utilities. | | | |  |  |  |  |  |  |
| ^b^ If each session has five people. | | | |  |  |  |  |  |  |
| ^c^ 25% decrease in building rental/utilities. | | | |  |  |  |  |  |  |
| ^d^ If each session has three people. | | | |  |  |  |  |  |  |
| ^e^ See Tables 5 and 6 for the values used here. Included in parentheses next to the clinically significant changes are the corresponding percentage changes. | | | | | | | | | |
| ^f^ Occasions = days used * times per day. | |  |  |  |  |  |  |  |  |
| ^g^ Varying base case values through clinically significant ranges and statistically significant switching points to assess changes in CEA threshold. | | | | | | | | | |
| ^h^ Proportion of patients abstaining from cocaine. | | | | | | | |  |  |
| ^i^ Varying base case values through ranges of statistically significant switching points to assess changes in CEA threshold. | | | | | | | | |  |
| ^j^ Proportion of patients abstaining from alcohol. | | | |  |  |  |  |  |  |
